# The application of metagenomic next-generation sequencing in pathogen diagnosis: a bibliometric analysis based on Web of Science

**DOI:** 10.3389/fcimb.2023.1112229

**Published:** 2023-08-03

**Authors:** Sike He, Jingwen Wei, Jiaming Feng, Dan Liu, Neng Wang, Liyu Chen, Ying Xiong

**Affiliations:** ^1^ West China School of Medicine, Sichuan University, Chengdu, China; ^2^ Department of Periodical Press, West China Hospital, Sichuan University, Chengdu, China; ^3^ Center of Infectious Diseases, West China Hospital, Sichuan University, Chengdu, China

**Keywords:** metagenomic next-generation sequencing, pathogen diagnosis, bibliometric analysis, Web of Science, VOSviewer, CiteSpace

## Abstract

**Background:**

Infectious disease is a large burden on public health globally. Metagenomic next-generation sequencing (mNGS) has become popular as a new tool for pathogen diagnosis with numerous advantages compared to conventional methods. Recently, research on mNGS increases yearly. However, no bibliometric analysis has systematically presented the full spectrum of this research field. Therefore, we reviewed all the publications associated with this topic and performed this study to analyze the comprehensive status and future hotspots of mNGS for infectious disease diagnosis.

**Methods:**

The literature was searched in the Web of Science Core Collection and screened without year or language restrictions, and the characteristics of the studies were also identified. The outcomes included publication years, study types, journals, countries, authorship, institutions, frontiers, and hotspots with trends. Statistical analysis and visualization were conducted using VOSviewer (version 1.6.16) and CiteSpace (version 6.1. R3).

**Results:**

In total, 325 studies were included in the analysis after screening. Studies were published between 2009 and 2022 with a significantly increasing number from 1 to 118. Most of the studies were original articles and case reports. *Frontiers in Cellular and Infection Microbiology* and *Clinical Infectious Disease* were the most commonly cited and co-cited journals. Institutions and researchers from China contributed the most to this field, followed by those from the USA. The hotspots and frontiers of these studies are pneumonia, tuberculosis, and central nervous system infections.

**Conclusion:**

This study determined that mNGS is a hot topic in the diagnosis of infectious diseases with development trends and provides insights into researchers, institutions, hotspots and frontiers in mNGS, which can offer references to related researchers and future research.

## 1 Introduction

As a series of common and frequently occurring diseases, infectious diseases have always been a heavy burden to global health due to their high morbidity and mortality rate, especially in developing countries (McArthur, 2019). First, some ancient infectious diseases, such as tuberculosis (TB) caused by mycobacterium tuberculosis (MTB), have plagued mankind for thousands of years, and a quarter of the global population is infected with MTB (Kherabi et al., 2022). Additionally, some explosive epidemics, including seasonal influenza and COVID-19, have caused great losses in health, finances, and emotions (Calabrò et al., 2022; Schäfer et al., 2022; Xie et al., 2022). Precise pathogen diagnosis is a key part of infectious disease management. However, rare pathogens, emerging infectious diseases, and the prevalence of drug resistance present huge challenges to the diagnosis and further management (Mancuso et al., 2021; Vitiello, 2022).

Etiology is the gold standard for infectious disease diagnosis (Esposito, 2016). The detection technology of the traditional etiological method is culture with high positive predictive value. However, the limitations, including narrow pathogen spectrum, low positive rate, and long detection cycle, are nonnegligible. These drawbacks may lead to delays in diagnosis, the irrational use of antibiotics, etc., which can result in poor prognosis (Carbo et al., 2021; Chen et al., 2022). Metagenomic next-generation sequencing (mNGS), based on high-throughput sequencing technology, is a burgeoning unbiased pathogen detection method (Chiu and Miller, 2019; Gu et al., 2019). mNGS mainly consists of nucleic acid extraction, library preparation, host sequence exclusion and pathogen sequence enrichment (Jia et al., 2021). It can characterize all DNA or RNA in samples and analyze the entire microbiome, human host genome, and transcriptome in clinical samples (Chiu and Miller, 2019). The advantages, such as high throughput, wide coverage, high accuracy, and efficiency have brought new light to clinical use. According to the study by Duan et al., mNGS presented significantly higher sensitivity than the traditional culture method (67.4% vs 23.6%, p < 0.001) and similar specificity (68.8% vs 81.3%; p =  0.41) (Duan et al., 2021). Furthermore, mNGS is more effective than the conventional method that it required 3 days to identify 67.23% of cases of tuberculosis (TB), whereas 49.58% detected using conventional methods required over 90 days (Shi et al., 2020). Additionally, it can markedly increase the detection rate for some rare pathogens with a low positive rate of culture or insufficient clinical precedents (Simner et al., 2018). Therefore, it has been successfully applied in the diagnosis and treatment of difficult and critical infectious diseases, the identification of unknown pathogens, drug resistance gene monitoring, and epidemiological tracking investigations (Chiu and Miller, 2019; Han et al., 2019).

Bibliometrics is a new cross-science approach that can analyze all knowledge carriers using mathematical and statistical methods (Blakeman, 2018). It has been widely used to guide researchers in specific fields via the quantitative research assessment of academic output to improve research efficiency (Tang et al., 2018; Yeung et al., 2018). Citation analysis is the core part of bibliometric analysis because the number of citations can reflect the impact of an article to a certain extent (Feijoo et al., 2014; Xiong et al., 2022). Many medical articles have explored certain research fields via bibliometric analysis have been published, including diabetes (Zhao et al., 2016), tuberculosis (Xiong et al., 2022), and vaccines(Zhang et al., 2019). With the widespread use of mNGS, the number of studies about this topic has increased in recent years. However, there is no study concerning the overview of mNGS in pathogen diagnosis based on bibliometric analysis. Thus, we performed this study to identify the current status, future hotspots, and frontiers of mNGS by analyzing the publications from core collection in Web of Science (WoSCC).

## 2 Methods

### 2.1 Data collection

We extracted literature from the Science Citation Index database in the WoSCC via the Sichuan University Library website. The search strategy was “(TI= (metagenomic next-generation sequencing)) OR TI= (mNGS),” and we downloaded all data within one day on October 1, 2022. There were no restrictions on language or publication time to ensure comprehensive search results. Two researchers (SKH and JMF) screened the literature by excluding meeting abstracts, letters, book chapters, corrections, and articles not related to this topic. If two investigators have a discrepancy, the third author (LYC) will be consulted.

### 2.2 Data extraction

Two researchers (SKH and DL) extracted data from all the included studies. The extracted data included the title, abstract, keywords, references, source journal, publication date, total citations of all databases, authors with affiliation, country, and journal impact factor (IF). The institutions and countries were counted based on the corresponding authors. Journal IFs were obtained from Journal Citation Reports (JCR) 2022 to reflect the academic influence. Two researchers (NW and LYC) from the Center of Infectious Diseases in West China Hospital analyzed and screened the keywords, excluding some words without significant relevance, and merged synonyms (e.g., pcr and PCR). The third investigator (LYC) will be consulted when divergence occurs.

### 2.3 Data analysis and visualization

Microsoft (version 2022) was used to organize the publications and analyze their basic characteristics. The number of publications and citations annually were plotted by Stata (version 16.0). Bibliometric visualization was completed by VOSviewer (version 1.6.16) and CiteSpace (version 6.1. R3), including the journals, authors, countries, affiliations, and keywords in this research field regarding the use of mNGS in pathogen detection. VOSviewer was applied in co-citation and co-occurrence analysis. In the collaborative network map, each node represented one element (i.e., journal, country, author), and the size of each circle was weighted using document numbers and the line in the visualization reflected the relatedness of links. In the density map of keywords com-occurrence analysis, the colors ranged from blue to green to yellow. Yellow areas represented research hotspots and directions in this field. The “author keywords” was set in co-occurrence analysis. CiteSpace was applied to track the trend of keywords. The significance test was not used for statistical analysis because no control was involved.

## 3 Results

### 3.1 General information of the included literature

**Figure 1:**
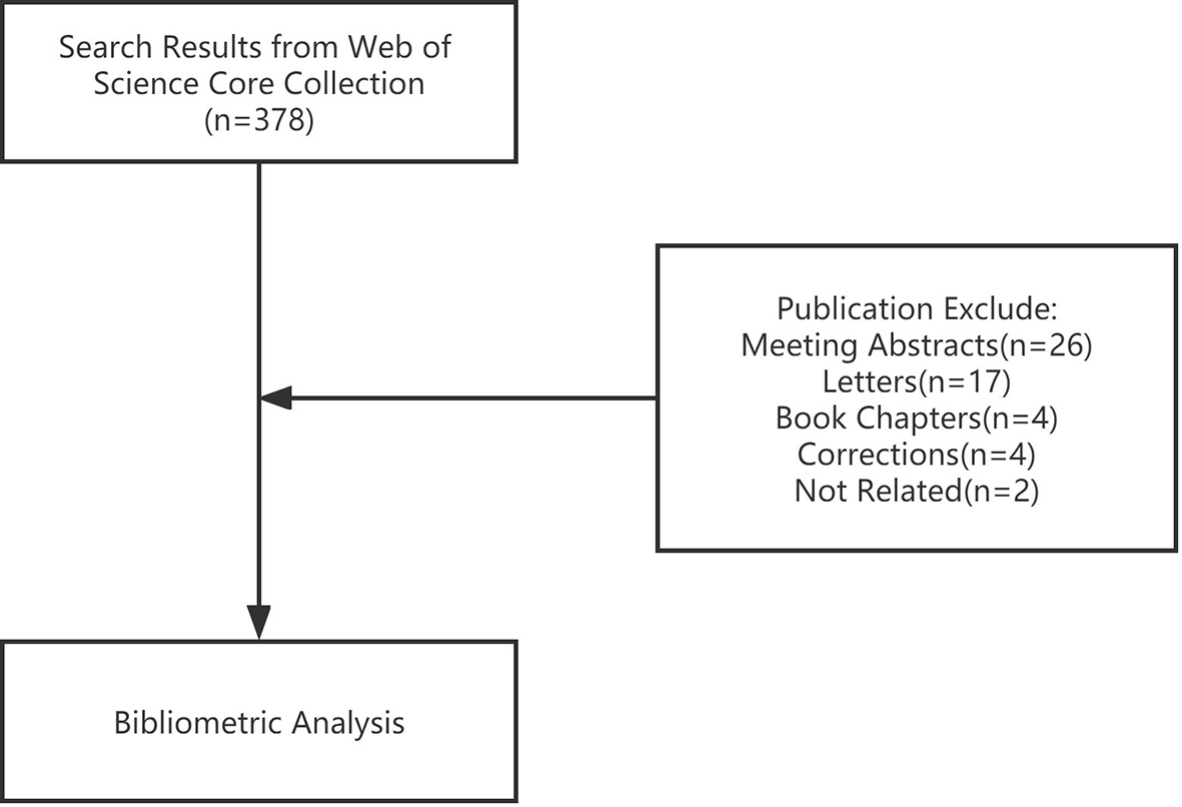
Flow chart of the study.

**Figure 2:**
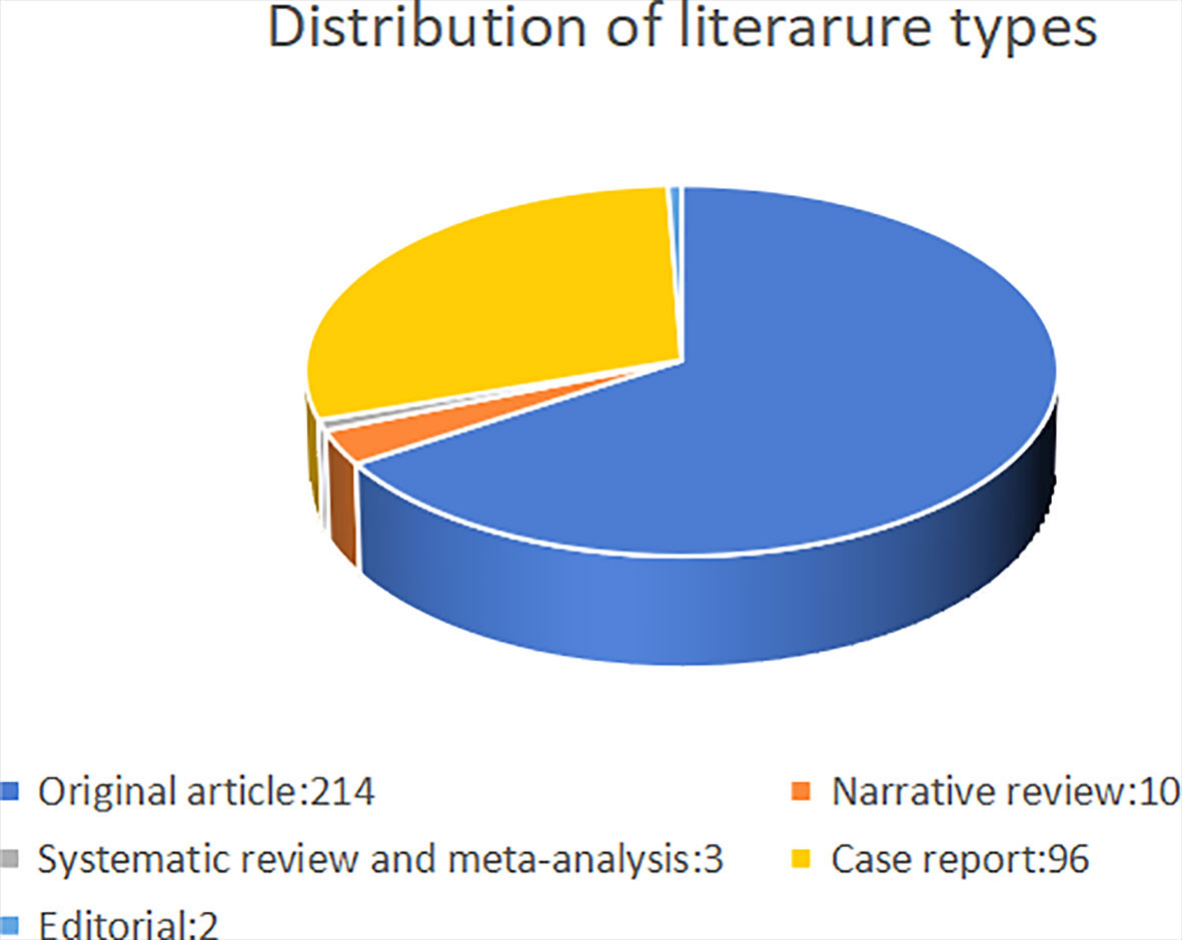
The distribution of the included studies.

**Figure 3:**
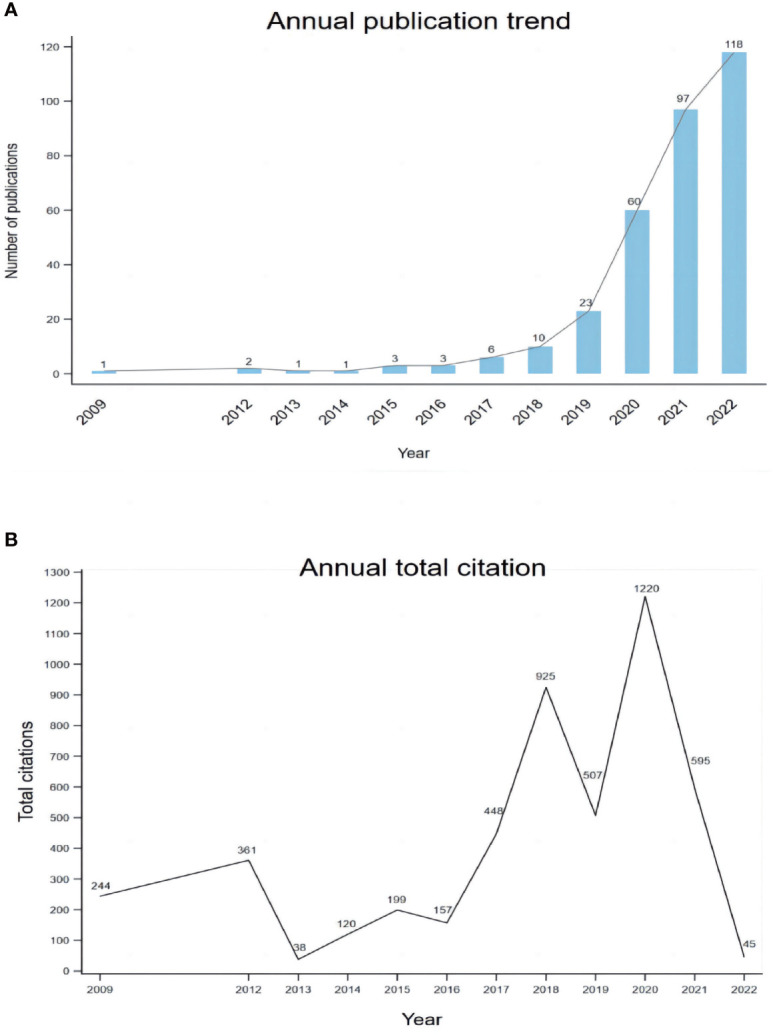
The trend of publications on mNGS in infectious diseases diagnosis from 2009 to 2022 **(A)** and the annual number of total citations **(B)**.

In total, 378 studies were conducted. After excluding meeting abstracts (26), letters (17), book chapters (4), corrections (4), and articles unrelated to the topic (2), we finally included 325 studies for further analysis (**Figure 1**). The study types (i.e., article, review, case report) were counted and the distribution is shown in **Figure 2**. Original article (n=214) and case report (n=96) accounted for the majority (65.8% and 29.5%). All the studies were published over 13 years from 2009 to 2022, and the number of publications increased during the past years, with an upwards trend (**Figure 3A**). The annual citation account was also conducted. From 2013 to 2020, the total citations increased significantly, which is consistent with the annual publications (**Figure 3B**). The top 20 most cited studies with details are displayed in **Table 1**.

**Table 1:** The top 20 most cited studies.

Title	First author	Corresponding author	Year	Journal	IF	Total citations
RNA based mNGS approach identifies a novel human coronavirus from two individual pneumonia cases in 2019 Wuhan outbreak	Liangjun Chen	Yingle Liu, Ke Lan, Mang Shi, Yirong Li, Yu Chen	2020	EMERG MICROBES INFEC	19.568	344
Microbiological Diagnostic Performance of Metagenomic Next-generation Sequencing When Applied to Clinical Practice	Miao Qing	Bijie Hu	2018	CLIN INFECT DIS	20.999	267
Understanding the Promises and Hurdles of Metagenomic Next-Generation Sequencing as a Diagnostic Tool for Infectious Diseases	Simner PJ	Simner PJ	2018	CLIN INFECT DIS	20.999	254
Next-generation sequencing and metagenomic analysis: a universal diagnostic tool in plant virology	Adams IP	Adams IP	2009	MOL PLANT PATHOL	5.520	244
Validation of Metagenomic Next-Generation Sequencing Tests for Universal Pathogen Detection	Schlaberg R	Schlaberg R	2017	ARCH PATHOL LAB MED	5.686	239
Next generation sequencing and bioinformatic bottlenecks: the current state of metagenomic data analysis	Scholz MB	Chain PS	2012	CURR OPIN BIOTECH	10.279	213
An ensemble strategy that significantly improves *de novo* assembly of microbial genomes from metagenomic next-generation sequencing data	Xutao Deng	Xutao Deng	2015	NUCLEIC ACIDS RES	19.190	168
Assessment of Metagenomic Assembly Using Simulated Next Generation Sequencing Data	Mende DR	Bork P	2012	PLOS ONE	3.752	148
Chronic Meningitis Investigated *via* Metagenomic Next-Generation Sequencing	Wilson MR	Wilson MR	2018	JAMA NEUROL	29.907	145
Comparison of three next-generation sequencing platforms for metagenomic sequencing and identification of pathogens in blood	Frey KG	Bishop-Lilly KA	2014	BMC GENOMICS	4.547	119
Rapid pathogen detection by metagenomic next-generation sequencing of infected body fluids	Wei GU	C.Y. Chiu	2021	NAT MED	87.241	116
Detection of Pulmonary Infectious Pathogens From Lung Biopsy Tissues by Metagenomic Next-Generation Sequencing	Henan Li	Hui Wang	2018	FRONT CELL INFECT MI	6.073	92
Coinfections of Zika and Chikungunya Viruses in Bahia, Brazil, Identified by Metagenomic Next-Generation Sequencing	Sardi SI	C.Y. Chiu	2016	J CLIN MICROBIOL	11.677	83
mNGS in clinical microbiology laboratories: on the road to maturity	Dongsheng Han	Jinming Li; Rui Zhang	2019	CRIT REV MICROBIOL	7.391	67
Rapid Metagenomic Next-Generation Sequencing during an Investigation of Hospital-Acquired Human Parainfluenza Virus 3 Infections	Greninger AL	Greninger AL	2017	J CLIN MICROBIOL	11.677	63
Neurobrucellosis: Unexpected Answer From Metagenomic Next-Generation Sequencing	Mongkolrattanothai K	C.Y. Chiu	2017	J PEDIATR INFECT DIS	5.235	60
Clinical Impact of Metagenomic Next-Generation Sequencing of Plasma Cell-Free DNA for the Diagnosis of Infectious Diseases: A Multicenter Retrospective Cohort Study	Hogan CA	Banaei N	2021	CLIN INFECT DIS	20.999	58
Metagenomic next-generation sequencing for mixed pulmonary infection diagnosis	Jiahui Wang	Jing Feng	2019	BMC PULM MED	3.320	57
Metagenomic Next-Generation Sequencing of Nasopharyngeal Specimens Collected from Confirmed and Suspect COVID-19 Patients	Mostafa HH	Simner PJ	2020	MBIO	7.786	57
Viral Surveillance in Serum Samples From Patients With Acute Liver Failure By Metagenomic Next-Generation Sequencing	Somasekar S	C.Y. Chiu	2017	CLIN INFECT DIS	20.999	54

### 3.2 Cited journals and co-cited journals

**Figure 4:**
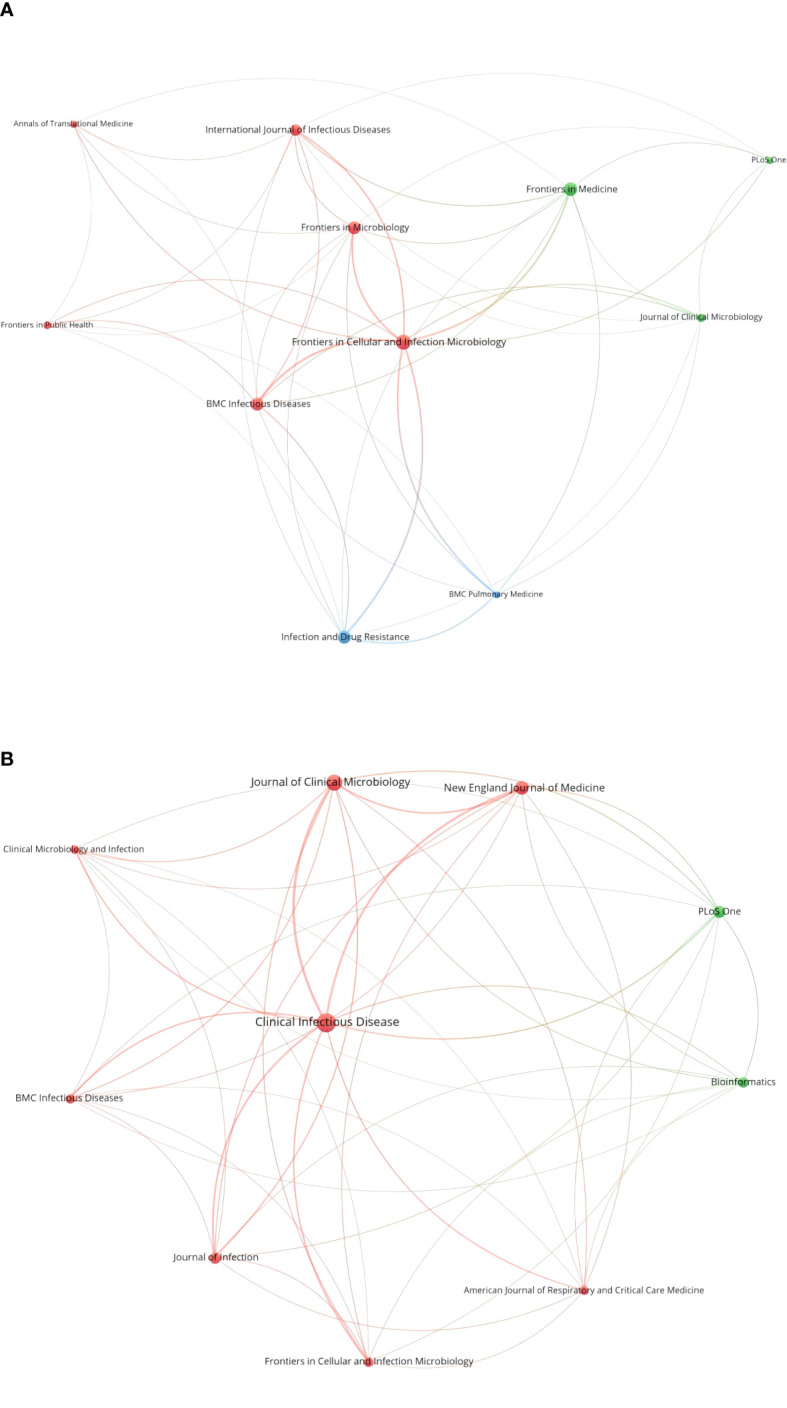
Collaborative network of the top 10 cited journals **(A)** and the top 10 co-cited journals **(B)**.

A total of 325 publications were published in 114 journals, and the top 10 cited journals are listed in **Table 2**. Most were published in *Frontiers in Cellular and Infection Microbiology* (n=28), followed by *Frontiers in Medicine* (n=22), *Infection and Drug Resistance* (n=21), *BMC Infectious Diseases* (n=20), *Frontiers in Microbiology* (n=20), *International Journal of Infectious Diseases* (n=15), *Journal of Clinical Microbiology* (n=9), *Frontiers in Public Health* (n=8), *Annals of Translational Medicine* (n=7), *BMC Pulmonary Medicine* (n=7), *and PLoS One* (n=7). The journals’ IFs ranged from 11.677 to 3.320. The collaborative network of journals is shown in **Figure 4A**.

**Table 2:** Top 10 cited and co-cited journals that published studies in this topic.

Cited Journal	Total citation	Impact factor (2022)	Co-cited journal	Total citation	Impact factor (2022)
*Frontiers in Cellular and Infection Microbiology*	222	6.073	*Clinical Infectious Disease*	604	20.999
*Frontiers in Medicine*	28	5.058	*Journal of Clinical Microbiology*	390	11.677
*Infection and Drug Resistance*	63	4.177	*New England Journal of Medicine*	288	176.079
*BMC Infectious Diseases*	146	3.667	*PLoS One*	241	3.752
*Frontiers in Microbiology*	140	6.064	*Journal of Infection*	203	38.637
*International Journal of Infectious Diseases*	115	12.074	*Bioinformatics*	181	6.931
*Journal of Clinical Microbiology*	276	11.677	*Frontiers in Cellular and Infection Microbiology*	162	6.073
*Annals of Translational Medicine*	19	3.616	*BMC Infectious Diseases*	159	3.667
*BMC Pulmonary Medicine*	81	3.320	*American Journal of Respiratory and Critical Care Medicine*	122	30.528
*PLoS One*	240	3.752	*Clinical Microbiology and Infection*	136	13.310

The top 10 co-cited journals are listed in **Table 2**. *Clinical Infectious Diseases* ranked first with 604 co-citations, followed by *Journal of Clinical Microbiology* (390), *New England Journal of Medicine* (288), *PLoS One* (241), and *Journal of Infection* (203). The network map of co-cited journals is shown in **Figure 4B**.

### 33 Distribution of contributing countries

**Figure 5:**
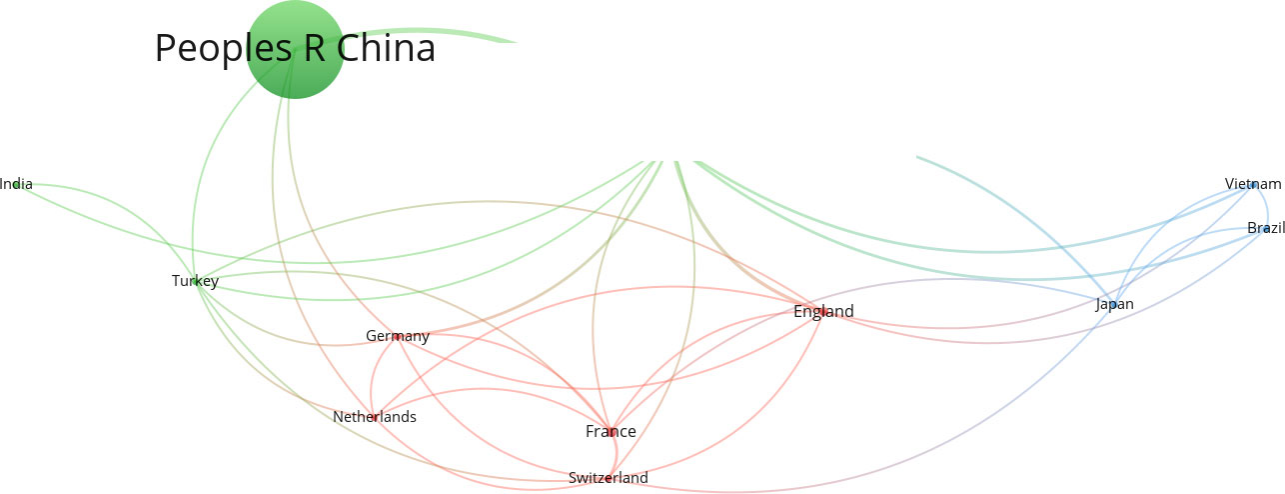
Collaborative network of the top 12 countries.

We list the top 11 countries based on the corresponding authors who published at least two studies (**Table 3**). China occupies the majority of the contributing countries (70%), followed by the USA and France, respectively. In addition, India, the Netherlands, Switzerland, Japan, Brazil, England, Vietnam, and Germany also contributed significantly to this field. According to the total citations, England ranked the first, followed by the USA, France, and China. The collaborative network of contributing countries is shown in **Figure 5**. The strength of the lines shows that the USA connected with most countries and had a great impact on their research. People R China and England follow the USA with similar strengths.

**Table 3:** Top 9 countries of the studies in mNGS (published at least 2 studies).

Country	Number of publication	Total citation	Average citation
People R China	249	2210	8.9
USA	43	1996	46.4
France	6	64	10.7
India	4	7	1.8
Netherlands	4	33	8.3
Japan	2	14	7
Switzerland	2	9	4.5
England	2	250	125
Vietnam	2	15	7.5

### 3.4 Distributions of institutions

**Figure 6:**
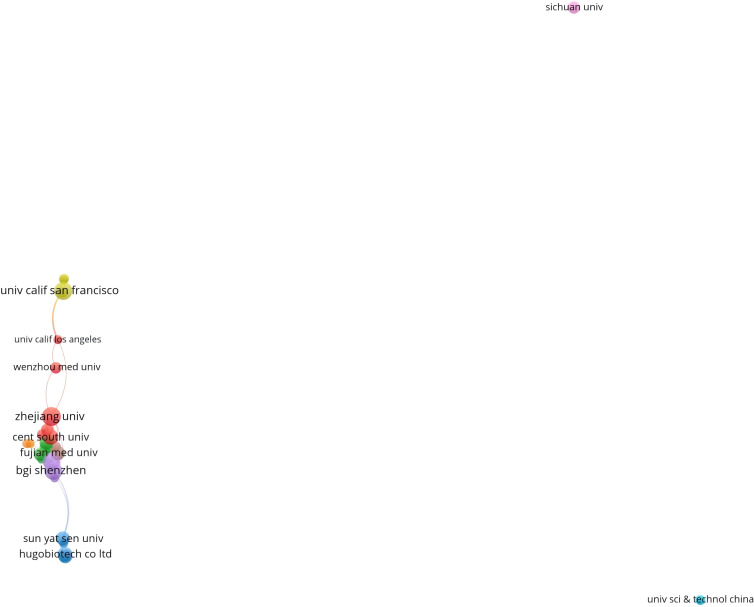
Collaborative network of institutions (published more than 5 articles).

In **Table 4**, the institutions with a large number of publications are listed. Eight institutions (based on the corresponding authors) contributed more than 10 studies, from People R China (7) and the USA (1). The top five institutions are Zhejiang University (20), Fudan University (11), Fujian Medical University (10), Tianjin Medical University (10), and Zhengzhou University (10). The collaborative network is displayed in **Figure 6**

**Table 4:** Top 8 institutions of corresponding authors (published at least 10 studies).

Institution	Country	Number of publication
Zhejiang University	People R China	20
Fudan University	People R China	14
Fujian Medical University	People R China	11
Tianjin Medical University	People R China	11
Zhengzhou University	People R China	11
Central South University	People R China	10
Sun Yat Sen University	People R China	10
University of California, San Diego	USA	10

### 3.5 Authors and co-cited authors

**Figure 7:**
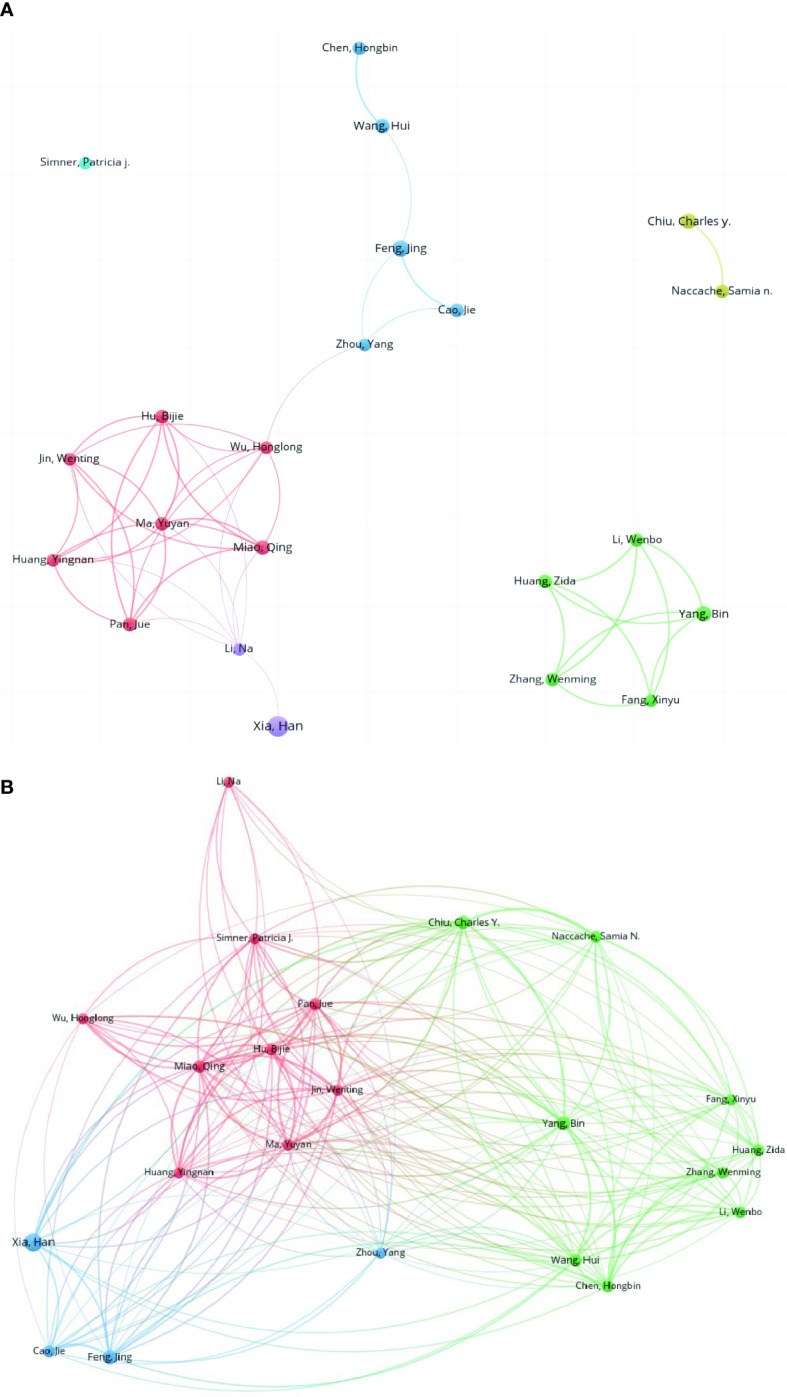
Collaborative network of the co-authorship **(A)** and co-citation relationships of authors **(B)**.

**Figure 7A** presents the collaborative network of authors (authors who have published more than five studies) in the publications. Most of them were from China. These authors are divided into groups with quite a few collaborative works. The top 22 authors with large numbers of publications (≥5) are listed in **Table 5**. Han Xia from Hugobiotech in China ranked first with 14 publications, followed by Jing Feng (9), Charles Y Chiu (8), Bin Yang (8), Qing Miao (7), Hui Wang (7), and so on. In **Figure 7B**, the co-citation network map of authors is presented.

**Table 5:** Top 22 corresponding authors of the publications (published at least 5 studies).

Name	Number of publications	Institution	Country
Han Xia	14	Hugobiotech	People R China
Jing Feng	9	Department of Ophthalmology, Beijing Chaoyang Hospital, Capital Medical University	People R China
Charles Y Chiu.	8	Department of Laboratory Medicine, University of California San Francisco	USA
Bin Yang	8	Department of Laboratory Medicine, The First Affiliated Hospital of Fujian Medical University	People R China
Qing Miao	7	Department of Infectious Diseases, Zhongshan Hospital, Fudan University	People R China
Hui Wang	7	Department of Ophthalmology, Beijing Chaoyang Hospital, Capital Medical University	People R China
Jie Cao	6	Department of Respiratory and Critical Care Medicine, Tianjin Medical University General Hospital	People R China
Hongbin Chen	6	Department of Clinical Laboratory, Peking University People’s Hospital	People R China
Bijie Hu	6	Department of Infectious Diseases, Zhongshan Hospital, Fudan University	People R China
Zida Huang	6	Department of Orthopaedic Surgery, The First Affiliated Hospital of Fujian Medical University	People R China
Wenbo Li	6	Department of Orthopaedic Surgery, The First Affiliated Hospital of Fujian Medical University	People R China
Yuyuan Ma	6	Department of Infectious Diseases, Zhongshan Hospital, Fudan University	People R China
Samia N Naccache.	6	Department of Laboratory Medicine, University of California, San Francisco	USA
Jue Pan	6	Department of Infectious Diseases, Zhongshan Hospital of Fudan University	People R China
Wenming Zhang	6	Department of Orthopaedic Surgery, The First Affiliated Hospital of Fujian Medical University	People R China
Xinyu Fang	5	Department of Orthopedic Surgery, First Affiliated Hospital of Fujian Medical University	People R China
Yingnan Huang	5	Department of Infectious Diseases, Zhongshan Hospital, Fudan University	People R China
Wenting Jin	5	Department of Infectious Diseases, Zhongshan Hospital, Fudan University	People R China
Na Li	5	Department of Respiratory and Critical Care Medicine, The Second Affiliated Hospital of Chongqing Medical University	People R China
Patricia J Simner	5	Department of Pathology, Johns Hopkins University School of Medicine	USA
Honglong Wu	5	BGI PathoGenesis Pharmaceutical Technology,	People R China
Yang Zhou	5	BGI PathoGenesis Pharmaceutical Technology	People R China

### 36 Keywords co-occurrence and clusters

**Figure 8:**
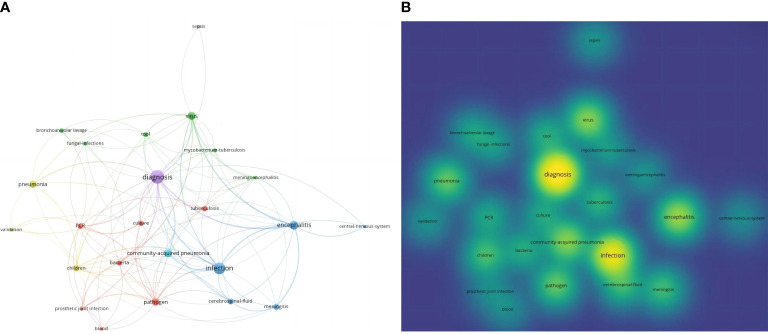
Cooccurrence network of key words **(A)** and cooccurrence density map of keywords **(B)**.

Twenty-four keywords were retained and their co-occurrence frequencies were calculated. The keyword co-occurrence network map is shown in **Figure 8A**. From the density map of keyword co-occurrence in **Figure 8B**, the keywords mainly focused on diagnosis, infection, encephalitis, community-acquired pneumonia, viruses, pathogens, pneumonia, children, cerebrospinal fluid, MTB, and meningitis.

### 3.7 Trend of keywords

**Figure 9:**
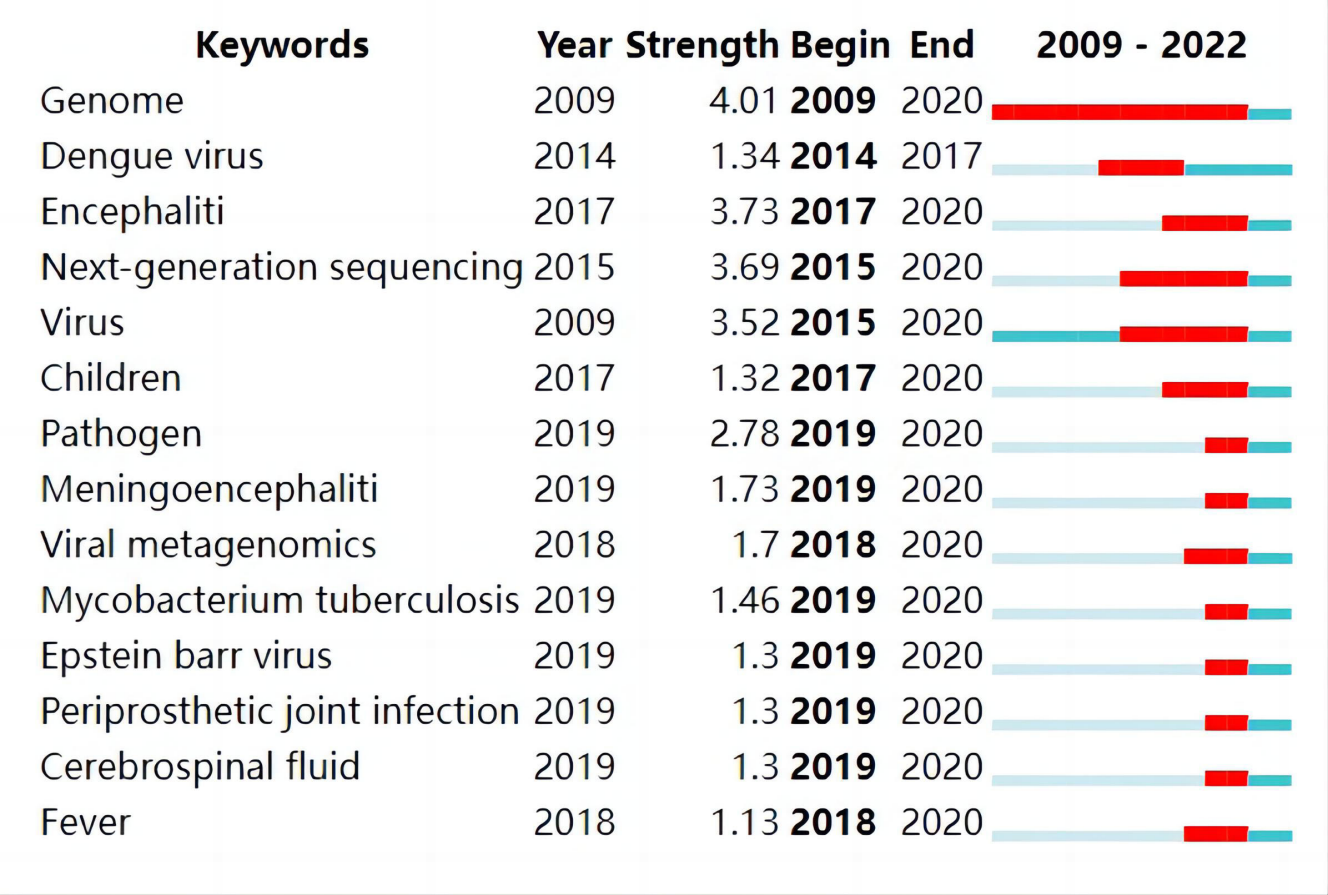
The trend of screened key words.

**Figure 9** presents the top 14 keywords with the strongest citation burst. “Genome”, “Encephalitis”, “Next-generation sequencing” and “Virus” were listed as the top 4 keywords with citation bursts of more than 3.50. The trend of some screened keywords and shows that some of them have been constantly heated thus far, such as genome and virus, while some of them have been focused on in recent years and can be considered as frontiers in the field, such as “children” (2017-2020), “meningoencephalitis” (2019-2020), “Mycobacterium tuberculosis (2019-2020)” and “cerebrospinal fluid (2019-2020)”. There are similarities between hotspots and frontiers.

## 4 Discussion

Pathogenic infection is a leading cause of disease and remains a severe problem for public health, which is involved in more than 20% of deaths per year globally (GBD 2019 Antimicrobial Resistance Collaborators, 2022). To accurately identify pathogens and precisely treat the disease, mNGS was initiated at the right moment. The utility of mNGS in providing clinically actionable information was first approved in a 14-year-old boy with *neuroleptospirosis* in 2014 (Wilson et al., 2014). At present, an increasing number of clinical cohort studies and case reports have confirmed the value of mNGS in pathogen diagnosis (Chiu and Miller, 2019; Li et al., 2021). However, the studies on mNGS are mostly comprised of case reports and cohort studies. To the best of our knowledge, this is the first bibliometric analysis focused on mNGS in pathogen detection.

### 4.1 General information

The number of publications significantly increased since 2014. After the first clinical use, a large number of cases have been diagnosed using the new tool, and as a result, more studies were published. In addition to pathogen diagnosis, the use of mNGS in other fields also contributed to the increasing number of publications, such as the surveillance of antimicrobial resistance in the food supply (Oniciuc et al., 2018), prediction of the cause of infection, and evaluation of risk (Gliddon et al., 2018; Langelier et al., 2018). The total citations had a similar trend to the publications. However, there are some fluctuations that may be caused by some extremely highly cited studies in certain years. The classification of the study types revealed that researchers mainly focus on the utility of mNGS in some notable cases and assess the diagnostic performance in some infectious diseases via cohort studies, such as TB and meningitis. With the large number of original articles, some reviews and meta-analysis will be published to summarize the development or efficacy of mNGS in the future.

The studies were mostly published in journals related to general and integrative medicine (*Frontiers in Medicine*, *Annals of Translational Medicine*) and infectious diseases (*Frontiers in Cellular and Infection Microbiology*, *Infection and Drug Resistance*, *BMC Infectious Diseases*). This illustrates that the application of mNGS is currently limited in infectious diseases now. *Frontiers in Cellular and Infection Microbiology* was the most popular cited journal, and also ranked 6^th^ in the co-cited journals, which means it is vital in this field. *Clinical Infectious Diseases* ranked first in co-cited journals with high IF (20.999). The average IF of the top 10 popular journals was 5.948 and that of the top 10 co-cited journals was 31.165. More articles can be published in more fields in the future based on these high-quality references.

Our study showed that the publications are from many countries. However, People R China and the USA showed such a powerful influence that publications from People R China accounted for approximately 70% of the total. This may be related to the epidemiology in China. Although China has achieved great economic, public health, and healthcare development after the Severe Acute Respiratory Syndrome outbreak in 2003, there are still various infectious diseases (Lu et al., 2016; Liu et al., 2018). Second, owing to the complex ecological environment, the spectrum of pathogens is broad and includes many rare pathogens. Since mNGS is effective for pathogen identification and can aid doctors in rapid and accurate diagnosis, it has attracted a great deal of attention with plenty of literature published in China. The USA is the birthplace of mNGS for pathogen detection and has lead to extensive cohort studies to examine the efficacy. Furthermore, the USA and People R China have a strong relationship in cooperation with other countries. It can be speculated that mNGS is widely clinically applied in China, and the basic molecular mechanism has been studied extensively in the USA. Additionally, according to the average citations, England ranked the top. The study published in 2009 by Adams et al. from England was the first study to detect pathogens using mNGS in the world with 244 citations, which lead to the highest average citations of England (Adams et al., 2009). With the effort of many countries, mNGS can be improved for better application.

Considering the research institutions, Zhejiang University, Fudan University, and BGI in China contributed significantly to this field, while the University of California San Francisco in the USA also played a key role. It can be concluded that collaboration among authors is relatively fixed and independent. This is possibly because mNGS is used for detecting rare pathogens, which are distributed regionally, such as *leishmaniasis* in the central and western regions of China (Tang et al., 2022), hepatic *echinococcosis* in cattle-producing areas, and Dengue fever in tropical areas (Ren et al., 2022). Furthermore, mNGS is still in the development stage and there is currently no deep cross-team collaboration at the moment, and different teams have different research focuses. However, the lack of communication and cooperation among institutions certainly limited the development in this field. Therefore, the exchanges of research findings and more cooperation are essential and the academic barriers should be reduced to promote the development of mNGS.

Based on the results of author analysis, authors from China occupy the vast majority, which is similar to the distribution of countries and institutions. Xia Han from Hugobiotech is the most productive with 14 publications, followed by Feng Jing from Beijing Chaoyang Hospital with 9. Chiu Charles from the University of California San Francisco is one the most influential researches in this field. He is the corresponding author of the first case report of mNGS by Wilson et al. which was published in *New England Journal of Medicine* as a milestone. Then, a clinical trial investigating the detection efficacy of mNGS in body fluids was published in *Nature Medicine* with a high IF (87.241) and citation (Gu et al., 2021).

### 4.2 Hotspots and frontiers

Keywords can reflect the core contents and topics of the studies. Through the keyword analysis, the results showed that pneumonia, tuberculosis, central nervous system (CNS) infection, and children had a high frequency or upwards trend.

#### 4.2.1 Pneumonia

Pneumonia is one of the most common disease manifestations in respiratory infection and the leading cause of death, especially community acquired pneumonia (Biscevic-Tokic et al., 2013; Wunderink and Waterer, 2017; Wang et al., 2022). Since a variety of pathogens can cause pneumonia and some of the symptoms are nonspecific, the detection rate of traditional culture combined with clinical experience is still low and ineffective (Schlaberg et al., 2017; Zhu et al., 2018). mNGS has shown better diagnostic efficiency for pneumonia and other respiratory system infections (Hogan et al., 2021; Zhan et al., 2021). Chen et al. demonstrated that mNGS had high diagnostic accuracy in bronchoalveolar lavage fluid (BALF) with 78% sensitivity and 77% specificity (Chen et al., 2022). In a retrospective study focused on mixed respiratory infection cases, compared with the conventional method, the sensitivity was significantly higher (97.2% vs 13.9%, p < 0.001). However, the specificity was the opposite (63.2% vs 94.7%; p = 0.07) (Wang et al., 2019). Besides, the prevalence of coronavirus disease 2019 (COVID-19) has also contributed to making pneumonia a hotspot in recent years. This novel coronavirus was identified by RNA-based mNGS and the genome was revealed to provide insights for future research (Chen et al., 2020a). Additionally, acute respiratory distress syndrome (ARDS) in severe pneumonia, such as COVID-19, is critical and rapidly progresses, resulting in high morality and poor prognosis for survivors (Griffiths et al., 2019). mNGS is confirmed to be a valuable tool to improve the management of ARDS and provide better quality of life for survivors (Zhang et al., 2020). In summary, mNGS is a promising technique to detect pathogens in pneumonia and other respiratory infections.

#### 4.2.2 Tuberculosis

Similar to pneumonia, TB caused by MTB is another hot topic in mNGS. MTB diagnosis has been a challenge for decades. Because of the specific biological properties of MTB, the positive rate of culture is low and takes a long time (Wang et al., 2020; Floyd et al., 2022). Thus, the diagnosis of TB is a problem that usually leads to TB-related death. mNGS was tested in pulmonary TB and showed outstanding performance in diagnosis with high sensitivity and specificity (Chen et al., 2020b). In addition, the multiorgan lesions also contribute to diagnosis difficulty, especially extrapulmonary TB (EPTB) (Suárez et al., 2019). Pang et al. revealed that the positive rate for EPTB by traditional tests was only 12.8% (Pang et al., 2019). A retrospective study in China assessed the clinical efficacy of mNGS in EPTB diagnosis and found that the positive rate was significantly higher than other routine methods (p < 0.001). Another meta-analysis also proved that mNGS is more effective than traditional tests in TB meningitis (Yu et al., 2020). However, the detection threshold and the efficacy in TB-related coinfection are still controversial and deserve further research (Shi et al., 2020). All of the evidence indicated that mNGS is a potential resolution for TB diagnosis and more research is necessary in the future.

#### 4.2.3 CNS infection

CNS infections are life-threatening, usually resulting in poor prognosis (Yu et al., 2022). Because of the lack of specificity in clinical presentation and cerebrospinal fluid (CSF) parameters, as well as the low pathogen content in CSF, diagnosis remains a challenge (Leonard, 2017; Yu et al., 2022). Although the utility of mNGS matures in some samples such as blood and BALF, it remains unclear whether mNGS has better diagnostic efficiency in CSF. A meta-analysis focused on mNGS for bacterial meningoencephalitis elucidated that the diagnostic efficacy is satisfactory with an estimated AUC (area under curve) of the summary receiver operating characteristic curve of 0.91 (Kanaujia et al., 2022). However, since available evidence is divided and limited, more cohort studies or meta-analysis are expected to eliminate the discrepancy and present more comprehensive and reliable conclusions (Chen et al., 2021).

#### 4.2.4 Children

For children or pediatrics, it is considered another frontier. Infectious disease is the leading cause of death in children, especially those under 5 years old. Because of the various pathogens, occult onset, atypical clinical symptoms, and rapid progression, pediatric infection is difficult to diagnose. Timely and accurate tools are necessary for the selection of effective medical interventions to improve the prognosis (Tao et al., 2022). Many studies elucidated that mNGS can provide useful information for suspected cases and identify infection or noninfection (Yan et al., 2021; Tao et al., 2022). Nowadays, large number of case report have demonstrated the successful use of mNGS in pediatric infection. However, the overall diagnostic performance has not been validated, which is worth illustrating in the future studies.

Summarily, all the hotspots and frontiers indicate that mNGS is a potential test and can be widely used.

### 4.3 Highlights and limitations

This bibliometric analysis presents the status of mNGS in pathogen detection, including journals, countries, contributors, institutions, research hotspots, and frontiers via data visualization, and the studies in this field can be rapidly accessed from the **Supplementary Material**. Scholars in the field can quickly understand the status by reading this study and contribute more to this field.

Our study has some limitations. First, the citation analysis was based on the Web of Science Core Collection, which might have missed some important literature indexed by other databases, such as Google Scholar and Scopus, resulting in biased results. Second, we used an accurate title search which means that a small number of publications that did not mention metagenomic next-generation sequencing may not have been included. Third, since bibliometrics includes several secondary studies (such as reviews), the keywords and research focus of the secondary research may be different from those of the original studies, which may also lead to bias. In addition, due to the purpose of the study, we can only describe the overview of this research field without a detailed mechanism.

## 5 Conclusion

In conclusion, this study determined that mNGS has become a current research hotspot and related research has grown exponentially. This study shows the research hotspots in mNGS, research institutions, and researchers of most related research that can provide useful references for workers in infectious diseases in the future.

## Data availability statement

The original contributions presented in the study are included in the article/**Supplementary Material**. Further inquiries can be directed to the corresponding authors.

## Author contributions

Conceptualization, YX. Methodology, SH, JW, JF. Software, SH, JW. Formal Analysis, YX, DL. Writing – Original Draft Preparation, SH. Writing – Review and Editing, YX, LC. All authors contributed to the article and approved the submitted version.

## Supplementary material

Supplementary Table 1 The 325 studies from the Web of Science Core Collection.
