# Supplementary material for: The application of metagenomic next-generation sequencing in pathogen diagnosis: a bibliometric analysis based on Web of Science
Source: Front Cell Infect Microbiol. 2023 Aug 3;13:1112229. doi: 10.3389/fcimb.2023.1112229 (PMC10434771; doi:10.3389/fcimb.2023.1112229)
Supplement: Supplementary Table 1 — The 325 studies from the Web of Science Core Collection. [file Table_1.docx]

| Article Title | Article Type | Source Title | DOI |
| --- | --- | --- | --- |
| Application of metagenomic next-generation sequencing technique for diagnosing a specific case of necrotizing meningoencephalitis caused by human herpesvirus 2 | CASE | OPEN LIFE SCIENCES | 10.1515/biol-2022-0464 |
| A case report on mixed pulmonary infection of Nocardia nova, Mycobacterium tuberculosis, and Aspergillus fumigatus based on metagenomic next-generation sequencing | CASE | FRONTIERS IN PUBLIC HEALTH | 10.3389/fpubh.2022.927338 |
| Multisite Pseudomonas aeruginosa Infections Detected by Metagenomic Next-Generation Sequencing in a Child with Aplastic Anemia: A Case Report | CASE | LABORATORY MEDICINE | 10.1093/labmed/lmab123 |
| Early detection of gram-negative bacteria using metagenomic next-generation sequencing in acute respiratory distress syndrome: A case report | CASE | EXPERIMENTAL AND THERAPEUTIC MEDICINE | 10.3892/etm.2022.11510 |
| Metagenomic next-generation sequencing for identifying pathogens in patients with rheumatic diseases and diffuse pulmonary lesions: A retrospective diagnostic study | ORIGINAL | FRONTIERS IN CELLULAR AND INFECTION MICROBIOLOGY | 10.3389/fcimb.2022.963611 |
| Metagenomic next-generation sequencing provides prognostic warning by identifying mixed infections in nocardiosis | ORIGINAL | FRONTIERS IN CELLULAR AND INFECTION MICROBIOLOGY | 10.3389/fcimb.2022.894678 |
| Circular Whole-Transcriptome Amplification (cWTA) and mNGS Screening Enhanced by a Group Testing Algorithm (mEGA) Enable High-Throughput and Comprehensive Virus Identification | ORIGINAL | MSPHERE | 10.1128/msphere.00332-22 |
| Metagenomic next-generation sequencing restores the diagnosis of a rare infectious complication of B cell depletion | CASE | EUROPEAN JOURNAL OF CLINICAL MICROBIOLOGY & INFECTIOUS DISEASES | 10.1007/s10096-022-04484-7 |
| Application of metagenomic next-generation sequencing in the diagnosis and treatment of recurrent urinary tract infection in kidney transplant recipients | ORIGINAL | FRONTIERS IN PUBLIC HEALTH | 10.3389/fpubh.2022.901549 |
| The application of metagenomic next-generation sequencing for detection of pathogens from dialysis effluent in peritoneal dialysis-associated peritonitis | ORIGINAL | PERITONEAL DIALYSIS INTERNATIONAL | 10.1177/08968608221117315 |
| Application Value of Metagenomics Next-Generation Sequencing (mNGS) in Detection of Mucormycosis after Chemotherapy in Childhood Acute Leukemia |  | EVIDENCE-BASED COMPLEMENTARY AND ALTERNATIVE MEDICINE | 10.1155/2022/7366432 |
| Tropheryma whipplei detection by metagenomic next-generation sequencing in bronchoalveolar lavage fluid: A cross-sectional study | ORIGINAL | FRONTIERS IN CELLULAR AND INFECTION MICROBIOLOGY | 10.3389/fcimb.2022.961297 |
| Metagenomic next-generation sequencing in diagnosing Pneumocystis jirovecii pneumonia: A case report | CASE | OPEN LIFE SCIENCES | 10.1515/biol-2022-0094 |
| Diagnosis by metagenomic next-generation sequencing of a Talaromyces marneffei bloodstream infection in an HIV-negative child: A case report | CASE | FRONTIERS IN PEDIATRICS | 10.3389/fped.2022.903617 |
| Diagnostic Value of Metagenomic Next-Generation Sequencing for Pulmonary Infection in Intensive Care Unit and Non-Intensive Care Unit Patients | ORIGINAL | FRONTIERS IN CELLULAR AND INFECTION MICROBIOLOGY | 10.3389/fcimb.2022.929856 |
| Clinical Value of Metagenomic Next-Generation Sequencing in Immunocompromised Patients with Sepsis | ORIGINAL | MEDICAL SCIENCE MONITOR | 10.12659/MSM.937041 |
| Metagenomic profiles of Dermacentor tick pathogens from across Mongolia, using next generation sequencing |  | FRONTIERS IN MICROBIOLOGY | 10.3389/fmicb.2022.946631 |
| Improving Suspected Pulmonary Infection Diagnosis by Bronchoalveolar Lavage Fluid Metagenomic Next-Generation Sequencing: a Multicenter Retrospective Study |  | MICROBIOLOGY SPECTRUM | 10.1128/spectrum.02473-21 |
| Potential clinical impact of metagenomic next-generation sequencing of plasma for cervical spine injury with sepsis in intensive care unit: A retrospective study | ORIGINAL | FRONTIERS IN CELLULAR AND INFECTION MICROBIOLOGY | 10.3389/fcimb.2022.948602 |
| Application of Metagenomic Next-Generation Sequencing in the Diagnosis of Pneumonia Caused by Chlamydia psittaci |  | MICROBIOLOGY SPECTRUM | 10.1128/spectrum.02384-21 |
| Plasma metagenomic next-generation sequencing of microbial cell-free DNA detects pathogens in patients with suspected infected pancreatic necrosis |  | BMC INFECTIOUS DISEASES | 10.1186/s12879-022-07662-2 |
| Application of metagenomic next-generation sequencing in the detection of pathogens in bronchoalveolar lavage fluid of infants with severe pneumonia after congenital heart surgery |  | FRONTIERS IN MICROBIOLOGY | 10.3389/fmicb.2022.954538 |
| Metagenomic next-generation sequencing-guided antimicrobial treatment versus conventional antimicrobial treatment in early severe community-acquired pneumonia among immunocompromised patients (MATESHIP): A study protocol |  | FRONTIERS IN MICROBIOLOGY | 10.3389/fmicb.2022.927842 |
| Pathogen Detection by Metagenomic Next-Generation Sequencing During Neutropenic Fever in Patients With Hematological Malignancies |  | OPEN FORUM INFECTIOUS DISEASES | 10.1093/ofid/ofac393 |
| Metagenomic next-generation sequencing for the diagnosis of pulmonary aspergillosis in non-neutropenic patients: a retrospective study |  | FRONTIERS IN CELLULAR AND INFECTION MICROBIOLOGY | 10.3389/fcimb.2022.925982 |
| Diagnostic performance of metagenomic next-generation sequencing in non-tuberculous mycobacterial pulmonary disease when applied to clinical practice |  | INFECTION | 10.1007/s15010-022-01890-z |
| The performance of detecting Mycobacterium tuberculosis complex in lung biopsy tissue by metagenomic next-generation sequencing |  | BMC PULMONARY MEDICINE | 10.1186/s12890-022-02079-8 |
| Diagnostic Performance of Metagenomic Next-Generation Sequencing in Pediatric Patients: A Retrospective Study in a Large Children's Medical Center | ORIGINAL | CLINICAL CHEMISTRY | 10.1093/clinchem/hvac067 |
| Pulmonary Cryptococcosis Diagnosed by Metagenomic Next-Generation Sequencing in a Young Patient With Normal Immune Function: A Case Report | CASE | FRONTIERS IN PUBLIC HEALTH | 10.3389/fpubh.2022.942282 |
| Utility of Metagenomic Next-Generation Sequencing for Etiological Diagnosis of Patients with Sepsis in Intensive Care Units |  | MICROBIOLOGY SPECTRUM | 10.1128/spectrum.00746-22 |
| Clinical evaluation of metagenomic next-generation sequencing for detecting pathogens in bronchoalveolar lavage fluid collected from children with community-acquired pneumonia |  | FRONTIERS IN MEDICINE | 10.3389/fmed.2022.952636 |
| Metagenomic Next-Generation Sequencing Reveals the Profile of Viral Infections in Kidney Transplant Recipients During the COVID-19 Pandemic |  | FRONTIERS IN PUBLIC HEALTH | 10.3389/fpubh.2022.888064 |
| Mycoplasma hominis Meningitis Diagnosed by Metagenomic Next-Generation Sequencing in a Preterm Newborn: a Case Report and Literature Review | CASE | LABORATORY MEDICINE | 10.1093/labmed/lmac078 |
| Clinical Metagenomic Next-Generation Sequencing for Diagnosis of Secondary Glaucoma in Patients With Cytomegalovirus-Induced Corneal Endotheliitis |  | FRONTIERS IN MICROBIOLOGY | 10.3389/fmicb.2022.940818 |
| Anncaliia algerae Microsporidiosis Diagnosed by Metagenomic Next-Generation Sequencing, China |  | EMERGING INFECTIOUS DISEASES | 10.3201/eid2807.212315 |
| Diagnostic Value and Clinical Application of mNGS for Post-Liver Transplantation Infection: A Cross-Sectional Study With Case Reports |  | FRONTIERS IN MICROBIOLOGY | 10.3389/fmicb.2022.919363 |
| Prediction of COVID-19 Based on Genomic Biomarkers of Metagenomic Next-Generation Sequencing Data Using Artificial Intelligence Technology |  | ERCIYES MEDICAL JOURNAL | 10.14744/etd.2022.00868 |
| Metagenomic next-generation sequencing for the diagnosis of Chlamydia psittaci pneumonia |  | CLINICAL RESPIRATORY JOURNAL | 10.1111/crj.13519 |
| Application of metagenomic next-generation sequencing in suspected intraocular infections |  | EUROPEAN JOURNAL OF OPHTHALMOLOGY | 10.1177/11206721221107311 |
| Clinical characteristics of 14 cases of severe Chlamydia psittaci pneumonia diagnosed by metagenomic next-generation sequencing A case series |  | MEDICINE | 10.1097/MD.0000000000029238 |
| Metagenomic Next-Generation Sequencing Versus Traditional Laboratory Methods for the Diagnosis and Treatment of Infection in Liver Transplantation |  | FRONTIERS IN CELLULAR AND INFECTION MICROBIOLOGY | 10.3389/fcimb.2022.886359 |
| Evaluation of Metagenomic and Targeted Next-Generation Sequencing Workflows for Detection of Respiratory Pathogens from Bronchoalveolar Lavage Fluid Specimens |  | JOURNAL OF CLINICAL MICROBIOLOGY | 10.1128/jcm.00526-22 |
| The Effectiveness of Metagenomic Next-Generation Sequencing in the Diagnosis of Prosthetic Joint Infection: A Systematic Review and Meta-Analysis |  | FRONTIERS IN CELLULAR AND INFECTION MICROBIOLOGY | 10.3389/fcimb.2022.875822 |
| The Application Value of Metagenomic and Whole-Genome Capture Next-Generation Sequencing in the Diagnosis and Epidemiological Analysis of Psittacosis |  | FRONTIERS IN CELLULAR AND INFECTION MICROBIOLOGY | 10.3389/fcimb.2022.872899 |
| Analysis of endophytic microbiome dataset from roots of black pepper (Piper nigrum L.) cultivated in the Central Highlands region, Vietnam using 16S rRNA gene metagenomic next-generation sequencing |  | DATA IN BRIEF | 10.1016/j.dib.2022.108108 |
| Application of metagenomic next-generation sequencing for the diagnosis of intracranial infection of Listeria monocytogenes |  | ANNALS OF TRANSLATIONAL MEDICINE | 10.21037/atm-22-2186 |
| Clinical application of metagenomic next-generation sequencing technology in the diagnosis and treatment of pulmonary infection pathogens: A prospective single-center study of 138 patients | ORIGINAL | JOURNAL OF CLINICAL LABORATORY ANALYSIS | 10.1002/jcla.24498 |
| Case Report: Metagenomic Next-Generation Sequencing Clinches the Diagnosis of Acute Q Fever and Verified by Indirect Immunofluorescence Assay | CASE | FRONTIERS IN MEDICINE | 10.3389/fmed.2022.846526 |
| Diagnosis of cytomegalovirus encephalitis using metagenomic next-generation sequencing of blood and cerebrospinal fluid: A case report | CASE | WORLD JOURNAL OF CLINICAL CASES | 10.12998/wjcc.v10.i14.4601 |
| Case Report: Therapeutic Strategy With Delayed Debridement for Culture-Negative Invasive Group A Streptococcal Infections Diagnosed by Metagenomic Next-Generation Sequencing |  | FRONTIERS IN PUBLIC HEALTH | 10.3389/fpubh.2022.899077 |
| The clinical application of metagenomic next-generation sequencing for detecting pathogens in bronchoalveolar lavage fluid: case reports and literature review | CASE | EXPERT REVIEW OF MOLECULAR DIAGNOSTICS | 10.1080/14737159.2022.2071607 |
| Diagnosis of infectious diseases in immunocompromised hosts using metagenomic next generation sequencing-based diagnostics |  | BLOOD REVIEWS | 10.1016/j.blre.2021.100906 |
| Multilaboratory assessment of metagenomic next-generation sequencing for unbiased microbe detection |  | JOURNAL OF ADVANCED RESEARCH | 10.1016/j.jare.2021.09.011 |
| METAGENOMIC NEXT-GENERATION SEQUENCING DETECTS PATHOGENS IN ENDOPHTHALMITIS PATIENTS |  | RETINA-THE JOURNAL OF RETINAL AND VITREOUS DISEASES | 10.1097/IAE.0000000000003406 |
| Diagnostic value of metagenomic next-generation sequencing of bronchoalveolar lavage fluid for the diagnosis of suspected pneumonia in immunocompromised patients |  | BMC INFECTIOUS DISEASES | 10.1186/s12879-022-07381-8 |
| Screening Biomarkers and Constructing a Predictive Model for Symptomatic Urinary Tract Infection and Asymptomatic Bacteriuria in Patients Undergoing Cutaneous Ureterostomy: A Metagenomic Next-Generation Sequencing Study |  | DISEASE MARKERS | 10.1155/2022/7056517 |
| Diagnostic accuracy of the metagenomic next-generation sequencing (mNGS) for detection of bacterial meningoencephalitis: a systematic review and meta-analysis |  | EUROPEAN JOURNAL OF CLINICAL MICROBIOLOGY & INFECTIOUS DISEASES | 10.1007/s10096-022-04445-0 |
| Risk Factors and Outcome of Sepsis in Traumatic Patients and Pathogen Detection Using Metagenomic Next-Generation Sequencing |  | CANADIAN JOURNAL OF INFECTIOUS DISEASES & MEDICAL MICROBIOLOGY | 10.1155/2022/2549413 |
| Performance of Metagenomic Next-Generation Sequencing for the Diagnosis of Cryptococcal Meningitis in HIV-Negative Patients |  | FRONTIERS IN CELLULAR AND INFECTION MICROBIOLOGY | 10.3389/fcimb.2022.831959 |
| A Retrospective Analysis of Metagenomic Next Generation Sequencing (mNGS) of Cerebrospinal Fluid from Patients with Suspected Encephalitis or Meningitis Infections |  | JOURNAL OF HEALTHCARE ENGINEERING | 10.1155/2022/5641609 |
| Metagenomic Next-Generation Sequencing vs. Traditional Pathogen Detection in the Diagnosis of Infection After Allogeneic Hematopoietic Stem Cell Transplantation in Children | ORIGINAL | FRONTIERS IN MICROBIOLOGY | 10.3389/fmicb.2022.868160 |
| A case report and literature review: diagnosis and treatment of human immunodeficiency virus coinfected with visceral leishmania by metagenomic next-generation sequencing in China | CASE | ANNALS OF TRANSLATIONAL MEDICINE | 10.21037/atm-22-1351 |
| Diagnosis of Coxiella burnetii infection via metagenomic next-generation sequencing: a case report |  | BMC INFECTIOUS DISEASES | 10.1186/s12879-022-07309-2 |
| Recurrent Pneumonia With Tuberculosis and Candida Co-infection Diagnosed by Metagenomic Next-Generation Sequencing: A Case Report and Literature Review |  | FRONTIERS IN MEDICINE | 10.3389/fmed.2022.755308 |
| Diagnostic Value of Bronchoalveolar Lavage Fluid Metagenomic Next-Generation Sequencing in Pneumocystis jirovecii Pneumonia in Non-HIV Immunosuppressed Patients |  | FRONTIERS IN CELLULAR AND INFECTION MICROBIOLOGY | 10.3389/fcimb.2022.872813 |
| Strongyloidiasis in a Patient Diagnosed by Metagenomic Next-Generation Sequencing: A Case Report | CASE | FRONTIERS IN MEDICINE | 10.3389/fmed.2022.835252 |
| Persistent Pulmonary Interstitial Emphysema With Respiratory Infection: A Clinicopathological Analysis of Six Cases and Detection of Infectious Pathogens by Metagenomic Next-Generation Sequencing (mNGS) |  | FRONTIERS IN PEDIATRICS | 10.3389/fped.2022.836276 |
| Evaluating the diagnostic value of using metagenomic next-generation sequencing on bronchoalveolar lavage fluid and tissue in infectious pathogens located in the peripheral lung field |  | ANNALS OF PALLIATIVE MEDICINE | 10.21037/apm-21-3474 |
| Comparison and development of a metagenomic next-generation sequencing protocol for combined detection of DNA and RNA pathogens in cerebrospinal fluid |  | BMC INFECTIOUS DISEASES | 10.1186/s12879-022-07272-y |
| Application of Metagenomic Next-Generation Sequencing in Mycobacterium tuberculosis Infection |  | FRONTIERS IN MEDICINE | 10.3389/fmed.2022.802719 |
| Application of metagenomic next-generation sequencing for bronchoalveolar lavage diagnostics in patients with lower respiratory tract infections |  | JOURNAL OF INTERNATIONAL MEDICAL RESEARCH | 10.1177/03000605221089795 |
| Diagnostic performance of the metagenomic next-generation sequencing in lung biopsy tissues in patients suspected of having a local pulmonary infection |  | BMC PULMONARY MEDICINE | 10.1186/s12890-022-01912-4 |
| Diagnosis of lung squamous cell carcinoma based on metagenomic Next-Generation Sequencing |  | BMC PULMONARY MEDICINE | 10.1186/s12890-022-01894-3 |
| Invasive Pulmonary Aspergillosis Diagnosis via Peripheral Blood Metagenomic Next-Generation Sequencing |  | FRONTIERS IN MEDICINE | 10.3389/fmed.2022.751617 |
| Diagnosis and Surveillance of Neonatal Infections by Metagenomic Next-Generation Sequencing |  | FRONTIERS IN MICROBIOLOGY | 10.3389/fmicb.2022.855988 |
| Optimization of Early Antimicrobial Strategies for Lung Transplant Recipients Based on Metagenomic Next-Generation Sequencing |  | FRONTIERS IN MICROBIOLOGY | 10.3389/fmicb.2022.839698 |
| Early Identification of Fungal and Mycobacterium Infections in Pulmonary Granulomas Using Metagenomic Next-Generation Sequencing on Formalin fixation and paraffin embedding tissue |  | EXPERT REVIEW OF MOLECULAR DIAGNOSTICS | 10.1080/14737159.2022.2052046 |
| Case Report: Diagnosis of Primary Klebsiella pneumoniae in Cervical Spine by Metagenomic Next-Generation Sequencing | CASE | FRONTIERS IN SURGERY | 10.3389/fsurg.2022.800396 |
| Editorial: mNGS for Fungal Pulmonary Infection Diagnostics |  | FRONTIERS IN CELLULAR AND INFECTION MICROBIOLOGY | 10.3389/fcimb.2022.864163 |
| Toward accurate diagnosis and surveillance of bacterial infections using enhanced strain-level metagenomic next-generation sequencing of infected body fluids |  | BRIEFINGS IN BIOINFORMATICS | 10.1093/bib/bbac004 |
| Pneumocystis Jirovecii Pneumonia Diagnosis via Metagenomic Next-Generation Sequencing |  | FRONTIERS IN MEDICINE | 10.3389/fmed.2022.812005 |
| Integration of Interleukin-6 Improves the Diagnostic Precision of Metagenomic Next-Generation Sequencing for Infection in Immunocompromised Children |  | FRONTIERS IN MICROBIOLOGY | 10.3389/fmicb.2022.819467 |
| Application of Metagenomic Next-Generation Sequencing in the Etiological Diagnosis of Infective Endocarditis During the Perioperative Period of Cardiac Surgery: A Prospective Cohort Study | CASE | FRONTIERS IN CARDIOVASCULAR MEDICINE | 10.3389/fcvm.2022.811492 |
| Synovial tuberculosis in wrist diagnosed based on metagenomic next-generation sequencing: A case report |  | EUROPEAN JOURNAL OF INFLAMMATION | 10.1177/20587392221075507 |
| mNGS for identifying pathogens in febrile neutropenic children with hematological diseases |  | INTERNATIONAL JOURNAL OF INFECTIOUS DISEASES | 10.1016/j.ijid.2021.12.335 |
| Comparative diagnostic utility of metagenomic next-generation sequencing, GeneXpert, modified Ziehl-Neelsen staining, and culture using cerebrospinal fluid for tuberculous meningitis: A multi-center, retrospective study in China | ORIGINAL | JOURNAL OF CLINICAL LABORATORY ANALYSIS | 10.1002/jcla.24307 |
| Case Report: Metagenomic Next-Generation Sequencing Can Contribute to the Diagnosis and Treatment of Disseminated Visceral Kaposi Sarcoma Following Allogeneic Haematopoietic Stem Cell Transplantation | CASE | FRONTIERS IN ONCOLOGY | 10.3389/fonc.2022.848976 |
| Detection of Japanese Encephalitis by Metagenomic Next-Generation Sequencing of Cerebrospinal Fluid: A Case Report and Literature Review | CASE | FRONTIERS IN CELLULAR NEUROSCIENCE | 10.3389/fncel.2022.856512 |
| Brain Abscess Caused by Nocardia farcinica and Diagnosed by Metagenomic Next-Generation Sequencing: A Case Report | CASE | FRONTIERS IN MEDICINE | 10.3389/fmed.2022.803554 |
| Diagnostic Significance of Metagenomic Next-Generation Sequencing for Community-Acquired Pneumonia in Southern China |  | FRONTIERS IN MEDICINE | 10.3389/fmed.2022.807174 |
| Metagenomic Next-Generation Sequencing for Diagnosing Infections in Lung Transplant Recipients: A Retrospective Study | ORIGINAL | TRANSPLANT INTERNATIONAL | 10.3389/ti.2022.10265 |
| Metagenomic Next-Generation Sequencing for the Microbiological Diagnosis of Abdominal Sepsis Patients |  | FRONTIERS IN MICROBIOLOGY | 10.3389/fmicb.2022.816631 |
| Longitudinal Monitoring of DNA Viral Loads in Transplant Patients Using Quantitative Metagenomic Next-Generation Sequencing |  | PATHOGENS | 10.3390/pathogens11020236 |
| Application of mNGS in the Etiological Analysis of Lower Respiratory Tract Infections and the Prediction of Drug Resistance |  | MICROBIOLOGY SPECTRUM |  |
| Clinical Impact of Plasma Metagenomic Next-generation Sequencing in a Large Pediatric Cohort |  | PEDIATRIC INFECTIOUS DISEASE JOURNAL | 10.1097/INF.0000000000003395 |
| Insights into the Unique Lung Microbiota Profile of Pulmonary Tuberculosis Patients Using Metagenomic Next-Generation Sequencing |  | MICROBIOLOGY SPECTRUM |  |
| Case Report: Community-Acquired Legionella gormanii Pneumonia in an Immunocompetent Patient Detected by Metagenomic Next-Generation Sequencing | CASE | FRONTIERS IN MEDICINE | 10.3389/fmed.2022.819425 |
| Metagenomic Next-Generation Sequencing vs. Traditional Microbiological Tests for Diagnosing Varicella-Zoster Virus Central Nervous System Infection |  | FRONTIERS IN PUBLIC HEALTH | 10.3389/fpubh.2021.738412 |
| Case Report: Metagenomic Next-Generation Sequencing for Diagnosis of Human Encephalitis and Endophthalmitis Caused by Pseudorabies Virus | CASE | FRONTIERS IN MEDICINE | 10.3389/fmed.2021.753988 |
| Detection of Nocardia by 16S Ribosomal RNA Gene PCR and Metagenomic Next-Generation Sequencing (mNGS) |  | FRONTIERS IN CELLULAR AND INFECTION MICROBIOLOGY | 10.3389/fcimb.2021.768613 |
| Application of mNGS in the Etiological Diagnosis of Thoracic and Abdominal Infection in Patients With End-Stage Liver Disease |  | FRONTIERS IN CELLULAR AND INFECTION MICROBIOLOGY | 10.3389/fcimb.2021.741220 |
| Mycobacterium chelonae Infection Identified by Metagenomic Next-Generation Sequencing as the Probable Cause of Acute Contained Rupture of a Biological Composite Graft-A Case Report | CASE | INTERNATIONAL JOURNAL OF MOLECULAR SCIENCES | 10.3390/ijms23010381 |
| Diagnosis of Mycoplasma hominis Meningitis with Metagenomic Next-Generation Sequencing: A Case Report | CASE | INFECTION AND DRUG RESISTANCE | 10.2147/IDR.S371771 |
| Metagenomic Next-Generation Sequencing in the Diagnosis of Infectious Fever During Myelosuppression Among Pediatric Patients with and Diseases |  | INFECTION AND DRUG RESISTANCE | 10.2147/IDR.S379582 |
| A Case Report of Infective Endocarditis with Failure of the Empirical Treatment-Q Fever Endocarditis Diagnosed by Metagenomic Next-Generation Sequencing | CASE | INFECTION AND DRUG RESISTANCE | 10.2147/IDR.S361969 |
| Diagnosis of severe Chlamydia psittaci pneumonia by metagenomic next-generation sequencing: 2 case reports | CASE | RESPIRATORY MEDICINE CASE REPORTS | 10.1016/j.rmcr.2022.101709 |
| Pneumonia Caused by Coinfection with Cytomegalovirus and Pneumocystis jirovecii in an HIV-Negative Infant Diagnosed by Metagenomic Next-Generation Sequencing |  | INFECTION AND DRUG RESISTANCE | 10.2147/IDR.S364241 |
| Comparison of Droplet Digital PCR and Metagenomic Next-Generation Sequencing Methods for the Detection of Human Herpesvirus 6B Infection Using Cell-Free DNA from Patients Receiving CAR-T and Hematopoietic Stem Cell Transplantation |  | INFECTION AND DRUG RESISTANCE | 10.2147/IDR.S379439 |
| Application Value of Metagenomic Next-Generation Sequencing for Bloodstream Infections in Pediatric Patients Under Intensive Care |  | INFECTION AND DRUG RESISTANCE | 10.2147/IDR.S357162 |
| A Paired Comparison of Plasma and Bronchoalveolar Lavage Fluid for Metagenomic Next-Generation Sequencing in Critically Ill Patients with Suspected Severe Pneumonia |  | INFECTION AND DRUG RESISTANCE | 10.2147/IDR.S374906 |
| Case Report and Literature Review: Disseminated Histoplasmosis Infection Diagnosed by Metagenomic Next-Generation Sequencing |  | INFECTION AND DRUG RESISTANCE | 10.2147/IDR.S371740 |
| Metagenomic Next-Generation Sequencing for the Diagnosis of Suspected Opportunistic Infections in People Living with HIV |  | INFECTION AND DRUG RESISTANCE | 10.2147/IDR.S350047 |
| A Case of Secondary Pulmonary Syphilis-The Utility of mNGS in Bronchoalveolar Lavage Fluid: A Case Report | CASE | INFECTION AND DRUG RESISTANCE | 10.2147/IDR.S373711 |
| Cystoisospora belli infection in an AIDS patient in China: Need for cautious interpretation of mNGS |  | INDIAN JOURNAL OF MEDICAL MICROBIOLOGY | 10.1016/j.ijmmb.2021.10.005 |
| The Diagnosis of Severe Fever with Thrombocytopenia Syndrome Using Metagenomic Next-Generation Sequencing: Case Report and Literature Review | CASE | INFECTION AND DRUG RESISTANCE | 10.2147/IDR.S345991 |
| The Value of Metagenomic Next-Generation Sequencing in Hematological Malignancy Patients with Febrile Neutropenia After Empiric Antibiotic Treatment Failure |  | INFECTION AND DRUG RESISTANCE | 10.2147/IDR.S364525 |
| Corynebacterium striatum Endocarditis After Renal Transplantation Confirmed by Metagenomic Next-Generation Sequencing: Case Report and Literature Review | CASE | INFECTION AND DRUG RESISTANCE | 10.2147/IDR.S376985 |
| Metagenomic Next-Generation Sequencing for Accurate Diagnosis of Acute HIV Infection with Aseptic Meningitis: A Case Report | CASE | INFECTION AND DRUG RESISTANCE | 10.2147/IDR.S361049 |
| Metagenomic Next-Generation Sequencing of Cerebrospinal Fluid for the Diagnosis of Cerebral Aspergillosis |  | FRONTIERS IN MICROBIOLOGY | 10.3389/fmicb.2021.787863 |
| -D-Glucan for Differential Diagnosis of Pneumocystis jirovecii Pneumonia and Pneumocystis jirovecii Colonisation |  | FRONTIERS IN CELLULAR AND INFECTION MICROBIOLOGY | 10.3389/fcimb.2021.784236 |
| The microbiological diagnostic performance of metagenomic next-generation sequencing in patients with sepsis |  | BMC INFECTIOUS DISEASES | 10.1186/s12879-021-06934-7 |
| The application value of metagenomic next-generation sequencing in children with invasive pneumococcal disease |  | TRANSLATIONAL PEDIATRICS | 10.21037/tp-21-533 |
| Fast and precise pathogen detection and identification of overlapping infection in patients with CUTI based on metagenomic next-generation sequencing A case report | CASE | MEDICINE | 10.1097/MD.0000000000027902 |
| A Rare Bird: Diagnosis of Psittacosis Meningitis by Clinical Metagenomic Next-Generation Sequencing |  | OPEN FORUM INFECTIOUS DISEASES | 10.1093/ofid/ofab555 |
| Metagenomic next-generation sequencing in the diagnosis of leptospirosis presenting as severe diffuse alveolar hemorrhage: a case report and literature review | CASE | BMC INFECTIOUS DISEASES | 10.1186/s12879-021-06923-w |
| mNGS helped diagnose scrub typhus presenting as a urinary tract infection with high D-dimer levels: a case report | CASE | BMC INFECTIOUS DISEASES | 10.1186/s12879-021-06889-9 |
| Etiology of granulomatous lobular mastitis based on metagenomic next-generation sequencing |  | INTERNATIONAL JOURNAL OF INFECTIOUS DISEASES | 10.1016/j.ijid.2021.10.019 |
| Metagenomic Next-Generation Sequencing for Infectious Disease Diagnosis: A Review of the Literature With a Focus on Pediatrics | REVIEW | JOURNAL OF THE PEDIATRIC INFECTIOUS DISEASES SOCIETY | 10.1093/jpids/piab104 |
| Metagenomic Next-Generation Sequencing for Diagnosis of Pediatric Meningitis and Encephalitis: A Review | REVIEW | JOURNAL OF THE PEDIATRIC INFECTIOUS DISEASES SOCIETY | 10.1093/jpids/piab067 |
| Metagenomic Next-Generation Sequencing (mNGS): SARS-CoV-2 as an Example of the Technology's Potential Pediatric Infectious Disease Applications |  | JOURNAL OF THE PEDIATRIC INFECTIOUS DISEASES SOCIETY | 10.1093/jpids/piab108 |
| Future Applications of Metagenomic Next-Generation Sequencing for Infectious Diseases Diagnostics |  | JOURNAL OF THE PEDIATRIC INFECTIOUS DISEASES SOCIETY | 10.1093/jpids/piab107 |
| Case Report: Fascioliasis Hepatica Precisely Diagnosed by Metagenomic Next-Generation Sequencing and Treated With Albendazole | CASE | FRONTIERS IN MEDICINE | 10.3389/fmed.2021.773145 |
| The Early Diagnosis of Scrub Typhus by Metagenomic Next-Generation Sequencing |  | FRONTIERS IN PUBLIC HEALTH | 10.3389/fpubh.2021.755228 |
| Bacterial and Fungal Infections Promote the Bone Erosion Progression in Acquired Cholesteatoma Revealed by Metagenomic Next-Generation Sequencing |  | FRONTIERS IN MICROBIOLOGY | 10.3389/fmicb.2021.761111 |
| Metagenomic Next-Generation Sequencing for the Diagnosis of Epstein-Barr Virus Pleurisy: A Case Report | CASE | IRANIAN JOURNAL OF PUBLIC HEALTH |  |
| Meningitis Caused by the Live Varicella Vaccine Virus: Metagenomic Next Generation Sequencing, Immunology Exome Sequencing and Cytokine Multiplex Profiling |  | VIRUSES-BASEL | 10.3390/v13112286 |
| The Application of Metagenomic Next-Generation Sequencing in Detection of Pathogen in Bronchoalveolar Lavage Fluid and Sputum Samples of Patients with Pulmonary Infection |  | COMPUTATIONAL AND MATHEMATICAL METHODS IN MEDICINE | 10.1155/2021/7238495 |
| Use of Metagenomic Next-Generation Sequencing in the Clinical Microbiology Laboratory A Forward, but Not an End-All |  | JOURNAL OF MOLECULAR DIAGNOSTICS | 10.1016/j.jmoldx.2021.09.003 |
| Metagenomic next-generation sequencing to identify pathogens and cancer in lung biopsy tissue |  | EBIOMEDICINE | 10.1016/j.ebiom.2021.103639 |
| The potential of metagenomic next-generation sequencing in diagnosis of spinal infection: a retrospective study | ORIGINAL | EUROPEAN SPINE JOURNAL | 10.1007/s00586-021-07026-5 |
| Diagnostic Value of Metagenomic Next Generation Sequencing for Ureaplasma urealyticum Infection: A Case Report | CASE | LABORATORY MEDICINE | 10.1093/labmed/lmab091 |
| A Retrospective Paired Comparison Between Untargeted Next Generation Sequencing and Conventional Microbiology Tests With Wisely Chosen Metagenomic Sequencing Positive Criteria | ORIGINAL | FRONTIERS IN MEDICINE | 10.3389/fmed.2021.686247 |
| A Microbial World: Could Metagenomic Next-Generation Sequencing Be Involved in Acute Respiratory Failure? |  | FRONTIERS IN CELLULAR AND INFECTION MICROBIOLOGY | 10.3389/fcimb.2021.738074 |
| Metagenomic Next-Generation Sequencing in the Diagnosis of HHV-1 Reactivation in a Critically Ill COVID-19 Patient: A Case Report | CASE | FRONTIERS IN MEDICINE | 10.3389/fmed.2021.715519 |
| The clinical value of valve metagenomic next-generation sequencing when applied to the etiological diagnosis of infective endocarditis |  | ANNALS OF TRANSLATIONAL MEDICINE | 10.21037/atm-21-2488 |
| Clinical Impact of Metagenomic Next-Generation Sequencing of Bronchoalveolar Lavage in the Diagnosis and Management of Pneumonia A Multicenter Prospective Observational Study | ORIGINAL | JOURNAL OF MOLECULAR DIAGNOSTICS | 10.1016/j.jmoldx.2021.06.007 |
| Application of metagenomic next-generation sequencing in the diagnosis of severe pneumonia caused by Chlamydia psittaci |  | BMC PULMONARY MEDICINE | 10.1186/s12890-021-01673-6 |
| Detection of Coccidioides posadasii in a patient with meningitis using metagenomic next-generation sequencing: a case report | CASE | BMC INFECTIOUS DISEASES | 10.1186/s12879-021-06661-z |
| A case of paraplegia due to asymptomatic varicella-zoster virus infection in AIDS patient unexpectedly diagnosed by CSF metagenomic next-generation sequencing | CASE | BMC INFECTIOUS DISEASES | 10.1186/s12879-021-06611-9 |
| Metagenomic Next Generation Sequencing in the Detection of Pathogens in Cerebrospinal Fluid of Patients After Alternative Donor Transplantation: A Feasibility Analysis | ORIGINAL | FRONTIERS IN CELLULAR AND INFECTION MICROBIOLOGY | 10.3389/fcimb.2021.720132 |
| Detection of Neoplasms by Metagenomic Next-Generation Sequencing of Cerebrospinal Fluid |  | JAMA NEUROLOGY | 10.1001/jamaneurol.2021.3088 |
| An accurate and exact clustering algorithm for next generation sequencing metagenomic sequences |  | MATHEMATICAL METHODS IN THE APPLIED SCIENCES | 10.1002/mma.7748 |
| The Diagnostic Value of Metagenomic Next-Generation Sequencing in Lower Respiratory Tract Infection |  | FRONTIERS IN CELLULAR AND INFECTION MICROBIOLOGY | 10.3389/fcimb.2021.694756 |
| Detection of aerobe-anaerobe mixed infection by metagenomic next-generation sequencing in an adult suffering from descending necrotizing mediastinitis |  | BMC INFECTIOUS DISEASES | 10.1186/s12879-021-06624-4 |
| Metagenomic Next-Generation Sequencing of Bloodstream Microbial Cell-Free Nucleic Acid in Children With Suspected Sepsis in Pediatric Intensive Care Unit |  | FRONTIERS IN CELLULAR AND INFECTION MICROBIOLOGY | 10.3389/fcimb.2021.665226 |
| Metagenomic next-generation sequencing for the early diagnosis of talaromycosis in HIV-uninfected patients: five cases report |  | BMC INFECTIOUS DISEASES | 10.1186/s12879-021-06551-4 |
| Clinical Application of Metagenomic Next-Generation Sequencing for Suspected Infections in Patients With Primary Immunodeficiency Disease |  | FRONTIERS IN IMMUNOLOGY | 10.3389/fimmu.2021.696403 |
| Usefulness of metagenomic next-generation sequencing in adenovirus 7-induced acute respiratory distress syndrome: A case report | CASE | WORLD JOURNAL OF CLINICAL CASES | 10.12998/wjcc.v9.i21.6067 |
| Metagenomic Next-Generation Sequencing Can Clinch Diagnosis of Non-Tuberculous Mycobacterial Infections: A Case Report | CASE | FRONTIERS IN MEDICINE | 10.3389/fmed.2021.679755 |
| Use of Metagenomic Next-Generation Sequencing to Identify Pathogens in Pediatric Osteoarticular Infections |  | OPEN FORUM INFECTIOUS DISEASES | 10.1093/ofid/ofab346 |
| Case Report: Metagenomic Next-Generation Sequencing in Diagnosis of Disseminated Tuberculosis of an Immunocompetent Patient |  | FRONTIERS IN MEDICINE | 10.3389/fmed.2021.687984 |
| Metagenomic Next-Generation Sequencing for the Diagnosis of Pneumocystis jirovecii Pneumonia in Non-HIV-Infected Patients: A Retrospective Study | ORIGINAL | INFECTIOUS DISEASES AND THERAPY | 10.1007/s40121-021-00482-y |
| Detection of Chlamydia psittaci in both blood and bronchoalveolar lavage fluid using metagenomic next-generation sequencing A case report | CASE | MEDICINE | 10.1097/MD.0000000000026514 |
| Diagnosing scrub typhus without eschar: a case report using metagenomic next-generation sequencing (mNGS) | CASE | ANNALS OF TRANSLATIONAL MEDICINE | 10.21037/atm-21-3015 |
| Case Report: About a Case of Hyperammonemia Syndrome Following Lung Transplantation: Could Metagenomic Next-Generation Sequencing Improve the Clinical Management? | CASE | FRONTIERS IN MEDICINE | 10.3389/fmed.2021.684040 |
| The epidemic of Q fever in 2018 to 2019 in Zhuhai city of China determined by metagenomic next-generation sequencing |  | PLOS NEGLECTED TROPICAL DISEASES | 10.1371/journal.pntd.0009520 |
| A case report of pyogenic liver abscess caused by hypervirulent Klebsiella pneumoniae diagnosed by metagenomic next-generation sequencing | CASE | JOURNAL OF INTERNATIONAL MEDICAL RESEARCH | 10.1177/03000605211032793 |
| Early Detection of Legionella pneumophila and Aspergillus by mNGS in a Critically Ill Patient With Legionella Pneumonia After Extracorporeal Membrane Oxygenation Treatment: Case Report and Literature Review | CASE | FRONTIERS IN MEDICINE | 10.3389/fmed.2021.686512 |
| Exploring the Clinical Utility of Metagenomic Next-Generation Sequencing in the Diagnosis of Pulmonary Infection |  | INFECTIOUS DISEASES AND THERAPY | 10.1007/s40121-021-00476-w |
| Case Report: Metagenomic Next-Generation Sequencing in Diagnosis of Legionella pneumophila Pneumonia in a Patient After Umbilical Cord Blood Stem Cell Transplantation | CASE | FRONTIERS IN MEDICINE | 10.3389/fmed.2021.643473 |
| Application of Metagenomic Next-Generation Sequencing to Diagnose Pneumocystis jirovecii Pneumonia in Kidney Transplantation Recipients |  | ANNALS OF TRANSPLANTATION | 10.12659/AOT.931059 |
| Clinical Evaluation of an Improved Metagenomic Next-Generation Sequencing Test for the Diagnosis of Bloodstream Infections |  | CLINICAL CHEMISTRY | 10.1093/clinchem/hvab061 |
| Metagenomic Next-Generation Sequencing for Pathogenic Diagnosis and Antibiotic Management of Severe Community-Acquired Pneumonia in Immunocompromised Adults |  | FRONTIERS IN CELLULAR AND INFECTION MICROBIOLOGY | 10.3389/fcimb.2021.661589 |
| Case Report: A Severe and Multi-Site Nocardia farcinica Infection Rapidly and Precisely Identified by Metagenomic Next-Generation Sequencing | CASE | FRONTIERS IN MEDICINE | 10.3389/fmed.2021.669552 |
| A Comparison of Blood Pathogen Detection Among Droplet Digital PCR, Metagenomic Next-Generation Sequencing, and Blood Culture in Critically Ill Patients With Suspected Bloodstream Infections |  | FRONTIERS IN MICROBIOLOGY | 10.3389/fmicb.2021.641202 |
| Clinical usefulness of metagenomic next-generation sequencing for the diagnosis of central nervous system infection in people living with HIV |  | INTERNATIONAL JOURNAL OF INFECTIOUS DISEASES | 10.1016/j.ijid.2021.04.057 |
| Direct Diagnosis of Echovirus 12 Meningitis Using Metagenomic Next Generation Sequencing |  | PATHOGENS | 10.3390/pathogens10050610 |
| Use of Plasma Metagenomic Next-generation Sequencing for Pathogen Identification in Pediatric Endocarditis |  | PEDIATRIC INFECTIOUS DISEASE JOURNAL | 10.1097/INF.0000000000003038 |
| Application of metagenomic next-generation sequencing in the diagnosis and treatment guidance of Pneumocystis jirovecii pneumonia in renal transplant recipients |  | EUROPEAN JOURNAL OF CLINICAL MICROBIOLOGY & INFECTIOUS DISEASES | 10.1007/s10096-021-04254-x |
| Gastrointestinal manifestations of Talaromyces marneffei infection in an HIV-infected patient rapidly verified by metagenomic next-generation sequencing: a case report | CASE | BMC INFECTIOUS DISEASES | 10.1186/s12879-021-06063-1 |
| Metagenomic next-generation sequencing for identifying pathogens in central nervous system complications after allogeneic hematopoietic stem cell transplantation |  | BONE MARROW TRANSPLANTATION | 10.1038/s41409-021-01243-8 |
| Recommendations for the introduction of metagenomic next-generation sequencing in clinical virology, part II: bioinformatic analysis and reporting |  | JOURNAL OF CLINICAL VIROLOGY | 10.1016/j.jcv.2021.104812 |
| Metagenomic next-generation sequencing for the diagnosis of suspected pneumonia in immunocompromised patients |  | JOURNAL OF INFECTION | 10.1016/j.jinf.2021.01.029 |
| Cerebrospinal Fluid Analysis for Viruses by Metagenomic Next-Generation Sequencing in Pediatric Encephalitis: Not Yet Ready for Prime Time? |  | JOURNAL OF CHILD NEUROLOGY | 10.1177/0883073820972232 |
| Haemophilus influenzae Meningitis Direct Diagnosis by Metagenomic Next-Generation Sequencing: A Case Report | CASE | PATHOGENS | 10.3390/pathogens10040461 |
| Case Report: Metagenomic Next-Generation Sequencing in Diagnosis of Talaromycosis of an Immunocompetent Patient |  | FRONTIERS IN MEDICINE | 10.3389/fmed.2021.656194 |
| Metagenomic next-generation sequencing clinches diagnosis of leishmaniasis |  | LANCET |  |
| Application of Metagenomic Next-Generation Sequencing in the Diagnosis of Pulmonary Infectious Pathogens From Bronchoalveolar Lavage Samples |  | FRONTIERS IN CELLULAR AND INFECTION MICROBIOLOGY | 10.3389/fcimb.2021.541092 |
| Metagenomic Next-Generation Sequencing for Pathogen Detection and Transcriptomic Analysis in Pediatric Central Nervous System Infections |  | OPEN FORUM INFECTIOUS DISEASES | 10.1093/ofid/ofab104 |
| Metagenomic next-generation sequencing (mNGS) for diagnostically challenging infectious diseases in patients with acute leukemia |  | BRAZILIAN JOURNAL OF INFECTIOUS DISEASES | 10.1016/j.bjid.2021.101548 |
| Case Report: Proven Diagnosis of Culture-Negative Chronic Disseminated Candidiasis in a Patient Suffering From Hematological Malignancy: Combined Application of mNGS and CFW Staining | CASE | FRONTIERS IN MEDICINE | 10.3389/fmed.2021.627166 |
| Rapid diagnosis of Talaromyces marneffei infection assisted by metagenomic next-generation sequencing in a HIV-negative patient |  | IDCASES | 10.1016/j.idcr.2021.e01055 |
| A proof-of-concept study of an automated solution for clinical metagenomic next-generation sequencing |  | JOURNAL OF APPLIED MICROBIOLOGY | 10.1111/jam.15003 |
| Metagenomic next-generation sequencing of radial ultrasound bronchoscopy-guided cocktail specimens as an efficient method for the diagnosis of focal pulmonary infections: a randomised study | ORIGINAL | ANNALS OF PALLIATIVE MEDICINE | 10.21037/apm-20-2578 |
| Metagenomic next-generation sequencing technology for detection of pathogens in blood of critically ill patients |  | INTERNATIONAL JOURNAL OF INFECTIOUS DISEASES | 10.1016/j.ijid.2020.11.166 |
| Clinical efficacy of metagenomic next-generation sequencing for rapid detection of Mycobacterium tuberculosis in smear-negative extrapulmonary specimens in a high tuberculosis burden area |  | INTERNATIONAL JOURNAL OF INFECTIOUS DISEASES | 10.1016/j.ijid.2020.11.165 |
| Improving Pulmonary Infection Diagnosis with Metagenomic Next Generation Sequencing |  | FRONTIERS IN CELLULAR AND INFECTION MICROBIOLOGY | 10.3389/fcimb.2020.567615 |
| Unmasking viral sequences by metagenomic next-generation sequencing in adult human blood samples during steroid-refractory/dependent graft-versus-host disease |  | MICROBIOME | 10.1186/s40168-020-00953-3 |
| Tuberculosis Diagnosis by Metagenomic Next-generation Sequencing on Bronchoalveolar Lavage Fluid: a cross-sectional analysis | ORIGINAL | INTERNATIONAL JOURNAL OF INFECTIOUS DISEASES | 10.1016/j.ijid.2020.12.063 |
| Clinical Impact of Metagenomic Next-Generation Sequencing of Plasma Cell-Free DNA for the Diagnosis of Infectious Diseases: A Multicenter Retrospective Cohort Study | ORIGINAL | CLINICAL INFECTIOUS DISEASES | 10.1093/cid/ciaa035 |
| The diagnostic value of metagenomic next-generation sequencing in infectious diseases |  | BMC INFECTIOUS DISEASES | 10.1186/s12879-020-05746-5 |
| The clinical significance of simultaneous detection of pathogens from bronchoalveolar lavage fluid and blood samples by metagenomic next-generation sequencing in patients with severe pneumonia |  | JOURNAL OF MEDICAL MICROBIOLOGY | 10.1099/jmm.0.001259 |
| Effects of viral infection and microbial diversity on patients with sepsis: A retrospective study based on metagenomic next-generation sequencing | ORIGINAL | WORLD JOURNAL OF EMERGENCY MEDICINE | 10.5847/wjem.j.1920-8642.2021.01.005 |
| Optimizing culture methods according to preoperative mNGS results can improve joint infection diagnosis |  | BONE & JOINT JOURNAL | 10.1302/0301-620x.103b1.bjj-2020-0771.r2 |
| Metagenomic next-generation sequencing in the family outbreak of psittacosis: the first reported family outbreak of psittacosis in China under COVID-19 |  | EMERGING MICROBES & INFECTIONS | 10.1080/22221751.2021.1948358 |
| Clin-mNGS: Automated Pipeline for Pathogen Detection from Clinical Metagenomic Data |  | CURRENT BIOINFORMATICS | 10.2174/1574893615999200608130029 |
| Clinical Analysis of Metagenomic Next-Generation Sequencing Confirmed Chlamydia psittaci Pneumonia: A Case Series and Literature Review |  | INFECTION AND DRUG RESISTANCE | 10.2147/IDR.S305790 |
| Metagenomic Next-Generation Sequencing Assists in the Diagnosis of Gardnerella vaginalis in Males with Pleural Effusion and Lung Infection: A Case Report and Literature Review | CASE | INFECTION AND DRUG RESISTANCE | 10.2147/IDR.S337248 |
| Sixteen cases of severe pneumonia caused by Chlamydia psittaci in South China investigated via metagenomic next-generation sequencing |  | JOURNAL OF MEDICAL MICROBIOLOGY | 10.1099/jmm.0.001456 |
| Co-Infection Pneumonia with Mycobacterium abscessus and Pneumocystis jiroveci in a Patient without HIV Infection Diagnosed by Metagenomic Next-Generation Sequencing |  | INFECTION AND DRUG RESISTANCE | 10.2147/IDR.S292768 |
| Metagenomic Next-Generation Sequencing for Pulmonary Fungal Infection Diagnosis: Lung Biopsy versus Bronchoalveolar Lavage Fluid |  | INFECTION AND DRUG RESISTANCE | 10.2147/IDR.S333818 |
| Comparison of the efficacy of metagenomic next-generation sequencing and Xpert MTB/RIF in the diagnosis of tuberculous meningitis |  | JOURNAL OF MICROBIOLOGICAL METHODS | 10.1016/j.mimet.2020.106124 |
| Metagenomic Next-Generation Sequencing in the Diagnosis of Deep Sternal Wound Infection After Cardiac Transplantation: A Case Report and Literature Review | CASE | HEART SURGERY FORUM | 10.1532/HSF98.20214169 |
| Application of metagenomic next-generation sequencing (mNGS) combined with rapid on-site cytological evaluation (ROSCE) for the diagnosis of Chlamydia psittaci pneumonia |  | INTERNATIONAL JOURNAL OF CLINICAL AND EXPERIMENTAL PATHOLOGY |  |
| Clinical Characteristics of Chronic Lung Abscess Associated with Parvimonas micra Diagnosed Using Metagenomic Next-Generation Sequencing |  | INFECTION AND DRUG RESISTANCE | 10.2147/IDR.S304569 |
| Diagnosis of Streptococcus suis Meningoencephalitis with metagenomic next-generation sequencing of the cerebrospinal fluid: a case report with literature review | CASE | BMC INFECTIOUS DISEASES | 10.1186/s12879-020-05621-3 |
| Metagenomic Next-Generation Sequencing of Cerebrospinal Fluid for the Diagnosis of External Ventricular and Lumbar Drainage-Associated Ventriculitis and Meningitis |  | FRONTIERS IN MICROBIOLOGY | 10.3389/fmicb.2020.596175 |
| Metagenomic next-generation sequencing identified Histoplasma capsulatum in the lung and epiglottis of a Chinese patient: A case report | CASE | INTERNATIONAL JOURNAL OF INFECTIOUS DISEASES | 10.1016/j.ijid.2020.09.038 |
| Diagnostic Value of Metagenomic Next-Generation Sequencing for the Detection of Pathogens in Bronchoalveolar Lavage Fluid in Ventilator-Associated Pneumonia Patients |  | FRONTIERS IN MICROBIOLOGY | 10.3389/fmicb.2020.599756 |
| Metagenomic next generation sequencing for the diagnosis of tuberculosis meningitis: A systematic review and meta-analysis |  | PLOS ONE | 10.1371/journal.pone.0243161 |
| Clinical Utility of In-house Metagenomic Next-generation Sequencing for the Diagnosis of Lower Respiratory Tract Infections and Analysis of the Host Immune Response |  | CLINICAL INFECTIOUS DISEASES | 10.1093/cid/ciaa1516 |
| Etiology of Severe Community-Acquired Pneumonia in Adults Based on Metagenomic Next-Generation Sequencing: A Prospective Multicenter Study | ORIGINAL | INFECTIOUS DISEASES AND THERAPY | 10.1007/s40121-020-00353-y |
| Rapid pathogen detection by metagenomic next-generation sequencing of infected body fluids |  | NATURE MEDICINE | 10.1038/s41591-020-1105-z |
| Pathogen determination from clinical abscess fluids using metagenomic next-generation sequencing |  | FOLIA MICROBIOLOGICA | 10.1007/s12223-020-00829-x |
| Metagenomic Next-Generation Sequencing of Nasopharyngeal Specimens Collected from Confirmed and Suspect COVID-19 Patients |  | MBIO | 10.1128/mBio.01969-20 |
| Plasma Metagenomic Next-Generation Sequencing Assay for Identifying Pathogens: a Retrospective Review of Test Utilization in a Large Children's Hospital |  | JOURNAL OF CLINICAL MICROBIOLOGY | 10.1128/JCM.00794-20 |
| The clinical value of metagenomic next-generation sequencing in the microbiological diagnosis of skin and soft tissue infections |  | INTERNATIONAL JOURNAL OF INFECTIOUS DISEASES | 10.1016/j.ijid.2020.09.007 |
| Pulmonary actinomycosis diagnosed by radial endobronchial ultrasound coupled with metagenomic next-generation sequencing: A case report and brief literature review | CASE | INTERNATIONAL JOURNAL OF INFECTIOUS DISEASES | 10.1016/j.ijid.2020.09.1418 |
| Clinical assessment of the utility of metagenomic next-generation sequencing in pediatric patients of hematology department |  | INTERNATIONAL JOURNAL OF LABORATORY HEMATOLOGY | 10.1111/ijlh.13370 |
| Optimal specimen type for accurate diagnosis of infectious peripheral pulmonary lesions by mNGS |  | BMC PULMONARY MEDICINE | 10.1186/s12890-020-01298-1 |
| Metagenomic data on the composition of bacterial communities in lake environment sediments for fish farming by next generation Illumina sequencing |  | DATA IN BRIEF | 10.1016/j.dib.2020.106228 |
| Detecting the presence of bacteria in low-volume preoperative aspirated synovial fluid by metagenomic next-generation sequencing |  | INTERNATIONAL JOURNAL OF INFECTIOUS DISEASES | 10.1016/j.ijid.2020.07.039 |
| Leishmaniasis Diagnosis via Metagenomic Next-Generation Sequencing |  | FRONTIERS IN CELLULAR AND INFECTION MICROBIOLOGY | 10.3389/fcimb.2020.528884 |
| The metagenomic next-generation sequencing in diagnosing central nervous system angiostrongyliasis: a case report | CASE | BMC INFECTIOUS DISEASES | 10.1186/s12879-020-05410-y |
| Pathogenic Detection by Metagenomic Next-Generation Sequencing in Osteoarticular Infections |  | FRONTIERS IN CELLULAR AND INFECTION MICROBIOLOGY | 10.3389/fcimb.2020.00471 |
| Metagenomic Next-Generation Sequencing Improves Diagnosis of Osteoarticular Infections From Abscess Specimens: A Multicenter Retrospective Study |  | FRONTIERS IN MICROBIOLOGY | 10.3389/fmicb.2020.02034 |
| Thermus thermophilus DNA can be used as internal control for process monitoring of clinical metagenomic next-generation sequencing of urine samples |  | JOURNAL OF MICROBIOLOGICAL METHODS | 10.1016/j.mimet.2020.106005 |
| Diagnostic accuracy of metagenomic next-generation sequencing for active tuberculosis in clinical practice at a tertiary general hospital |  | ANNALS OF TRANSLATIONAL MEDICINE | 10.21037/atm-20-2274 |
| Application of metagenomic next-generation sequencing technology for difficult lung lesions in patients with haematological diseases |  | TRANSLATIONAL CANCER RESEARCH | 10.21037/tcr-20-604 |
| Metagenomic next-generation sequencing of viruses, bacteria, and fungi in the epineurium of the facial nerve with Bell's palsy patients |  | JOURNAL OF NEUROVIROLOGY | 10.1007/s13365-020-00892-7 |
| Blood and Bronchoalveolar Lavage Fluid Metagenomic Next-Generation Sequencing in Pneumonia |  | CANADIAN JOURNAL OF INFECTIOUS DISEASES & MEDICAL MICROBIOLOGY | 10.1155/2020/6839103 |
| Enhanced Detection of DNA Viruses in the Cerebrospinal Fluid of Encephalitis Patients Using Metagenomic Next-Generation Sequencing |  | FRONTIERS IN MICROBIOLOGY | 10.3389/fmicb.2020.01879 |
| Metagenomic next-generation sequencing of rectal swabs for the surveillance of antimicrobial-resistant organisms on the Illumina Miseq and Oxford MinION platforms |  | EUROPEAN JOURNAL OF CLINICAL MICROBIOLOGY & INFECTIOUS DISEASES | 10.1007/s10096-020-03996-4 |
| Acute viral encephalitis associated with human parvovirus B19 infection: unexpectedly diagnosed by metagenomic next-generation sequencing |  | JOURNAL OF NEUROVIROLOGY | 10.1007/s13365-020-00885-6 |
| Comparison of metagenomic next-generation sequencing technology, culture and GeneXpert MTB/RIF assay in the diagnosis of tuberculosis |  | JOURNAL OF THORACIC DISEASE | 10.21037/jtd-20-1232 |
| A case of pediatric visceral leishmaniasis-related hemophagocytic lymphohistiocytosis diagnosed by mNGS |  | INTERNATIONAL JOURNAL OF INFECTIOUS DISEASES | 10.1016/j.ijid.2020.05.056 |
| Metagenomic Characterization of Indoor Dust Bacterial and Fungal Microbiota in Homes of Asthma and Non-asthma Patients Using Next Generation Sequencing |  | FRONTIERS IN MICROBIOLOGY | 10.3389/fmicb.2020.01671 |
| Metagenomic next-generation sequencing for the clinical diagnosis and prognosis of acute respiratory distress syndrome caused by severe pneumonia: a retrospective study | ORIGINAL | PEERJ | 10.7717/peerj.9623 |
| Identification of Enterococcus faecalis in a patient with urinary-tract infection based on metagenomic next-generation sequencing: a case report | CASE | BMC INFECTIOUS DISEASES | 10.1186/s12879-020-05179-0 |
| Metagenomic next generation sequencing improves diagnosis of prosthetic joint infection by detecting the presence of bacteria in periprosthetic tissues |  | INTERNATIONAL JOURNAL OF INFECTIOUS DISEASES | 10.1016/j.ijid.2020.05.125 |
| Metagenomic next-generation sequencing of synovial fluid demonstrates high accuracy in prosthetic joint infection diagnostics |  | BONE & JOINT RESEARCH | 10.1302/2046-3758.97.BJR-2019-0325.R2 |
| Assessment of the Clinical Utility of Plasma Metagenomic Next-Generation Sequencing in a Pediatric Hospital Population |  | JOURNAL OF CLINICAL MICROBIOLOGY | 10.1128/JCM.00419-20 |
| Metagenomic Next -Generation Sequencing (mNGS) in cerebrospinal fluid for rapid diagnosis of Tuberculosis meningitis in HIV-negative population |  | INTERNATIONAL JOURNAL OF INFECTIOUS DISEASES | 10.1016/j.ijid.2020.04.048 |
| Clinical diagnostic application of metagenomic next-generation sequencing in children with severe nonresponding pneumonia |  | PLOS ONE | 10.1371/journal.pone.0232610 |
| Comparison of broad-range polymerase chain reaction and metagenomic next-generation sequencing for the diagnosis of prosthetic joint infection |  | INTERNATIONAL JOURNAL OF INFECTIOUS DISEASES | 10.1016/j.ijid.2020.03.055 |
| Clinical application and evaluation of metagenomic next-generation sequencing in suspected adult central nervous system infection |  | JOURNAL OF TRANSLATIONAL MEDICINE | 10.1186/s12967-020-02360-6 |
| Metagenomic Diagnosis for a Culture-Negative Sample From a Patient With Severe Pneumonia by Nanopore and Next-Generation Sequencing |  | FRONTIERS IN CELLULAR AND INFECTION MICROBIOLOGY | 10.3389/fcimb.2020.00182 |
| Metagenomic next-generation sequencing in the diagnosis of severe pneumonias caused by Chlamydia psittaci |  | INFECTION | 10.1007/s15010-020-01429-0 |
| Leprosy in a low-incidence setting Case report relevant to metagenomic next generation sequencing applications |  | WIENER KLINISCHE WOCHENSCHRIFT | 10.1007/s00508-020-01644-7 |
| The Value of Combined Radial Endobronchial Ultrasound-Guided Transbronchial Lung Biopsy and Metagenomic Next-Generation Sequencing for Peripheral Pulmonary Infectious Lesions |  | CANADIAN RESPIRATORY JOURNAL | 10.1155/2020/2367505 |
| Can metagenomic next-generation sequencing identify the pathogens responsible for culture-negative prosthetic joint infection? |  | BMC INFECTIOUS DISEASES | 10.1186/s12879-020-04955-2 |
| The application of metagenomic next-generation sequencing in diagnosing Chlamydia psittaci pneumonia: a report of five cases |  | BMC PULMONARY MEDICINE | 10.1186/s12890-020-1098-x |
| Metagenomic Next-Generation Sequencing for Diagnosis of Infectious Encephalitis and Meningitis: A Large, Prospective Case Series of 213 Patients |  | FRONTIERS IN CELLULAR AND INFECTION MICROBIOLOGY | 10.3389/fcimb.2020.00088 |
| A rare case of adrenal gland abscess due to anaerobes detected by metagenomic next-generation sequencing |  | ANNALS OF TRANSLATIONAL MEDICINE | 10.21037/atm.2020.01.123 |
| Point-Counterpoint: Should We Be Performing Metagenomic Next-Generation Sequencing for Infectious Disease Diagnosis in the Clinical Laboratory? |  | JOURNAL OF CLINICAL MICROBIOLOGY | 10.1128/JCM.01739-19 |
| Performance of Metagenomic Next-Generation Sequencing for the Diagnosis of Viral Meningoencephalitis in a Resource-Limited Setting |  | OPEN FORUM INFECTIOUS DISEASES | 10.1093/ofid/ofaa046 |
| Fulminant central nervous system varicella-zoster virus infection unexpectedly diagnosed by metagenomic next-generation sequencing in an HIV-infected patient: a case report | CASE | BMC INFECTIOUS DISEASES | 10.1186/s12879-020-4872-8 |
| Application of metagenomic next-generation sequencing for bronchoalveolar lavage diagnostics in critically ill patients |  | EUROPEAN JOURNAL OF CLINICAL MICROBIOLOGY & INFECTIOUS DISEASES | 10.1007/s10096-019-03734-5 |
| Reduction of Human DNA Contamination in Clinical Cerebrospinal Fluid Specimens Improves the Sensitivity of Metagenomic Next-Generation Sequencing |  | JOURNAL OF MOLECULAR NEUROSCIENCE | 10.1007/s12031-019-01472-z |
| Metagenomic Next-Generation Sequencing in Diagnosis of a Case of Pneumocystis jirovecii Pneumonia in a Kidney Transplant Recipient and Literature Review |  | INFECTION AND DRUG RESISTANCE | 10.2147/IDR.S257587 |
| RNA based mNGS approach identifies a novel human coronavirus from two individual pneumonia cases in 2019 Wuhan outbreak |  | EMERGING MICROBES & INFECTIONS | 10.1080/22221751.2020.1725399 |
| An unusual case report of brain abscess caused by prevotella loescheii identified using the metagenomic next-generation sequencing |  | IDCASES | 10.1016/j.idcr.2020.e00758 |
| Metagenomic Next-Generation Sequencing versus Traditional Pathogen Detection in the Diagnosis of Peripheral Pulmonary Infectious Lesions |  | INFECTION AND DRUG RESISTANCE | 10.2147/IDR.S235182 |
| Diagnosis of complication in lung transplantation by TBLB plus ROSE plus mNGS |  | OPEN MEDICINE | 10.1515/med-2020-0232 |
| Metagenomic next-generation sequencing helped diagnose scrub typhus without eschar: A case report | CASE | INTERNATIONAL JOURNAL OF INFECTIOUS DISEASES | 10.1016/j.ijid.2019.10.020 |
| Metagenomic next-generation sequencing for mixed pulmonary infection diagnosis |  | BMC PULMONARY MEDICINE | 10.1186/s12890-019-1022-4 |
| Culture-Negative Streptococcus suis Infection Diagnosed by Metagenomic Next-Generation Sequencing |  | FRONTIERS IN PUBLIC HEALTH | 10.3389/fpubh.2019.00379 |
| Metagenomic Next-Generation Sequencing for Identification and Quantitation of Transplant-Related DNA Viruses |  | JOURNAL OF CLINICAL MICROBIOLOGY | 10.1128/JCM.01113-19 |
| Progressive Multifocal Leukoencephalopathy Diagnosed by Metagenomic Next-Generation Sequencing of Cerebrospinal Fluid in an HIV Patient |  | FRONTIERS IN NEUROLOGY | 10.3389/fneur.2019.01202 |
| Incremental value of metagenomic next generation sequencing for the diagnosis of suspected focal infection in adults |  | JOURNAL OF INFECTION | 10.1016/j.jinf.2019.08.012 |
| mNGS in clinical microbiology laboratories: on the road to maturity |  | CRITICAL REVIEWS IN MICROBIOLOGY | 10.1080/1040841X.2019.1681933 |
| Clinical Evaluation of Diagnosis Efficacy of Active Mycobacterium tuberculosis Complex Infection via Metagenomic Next-Generation Sequencing of Direct Clinical Samples |  | FRONTIERS IN CELLULAR AND INFECTION MICROBIOLOGY | 10.3389/fcimb.2019.00351 |
| Arthritis caused by Legionella micdadei and Staphylococcus aureus: metagenomic next-generation sequencing provides a rapid and accurate access to diagnosis and surveillance |  | ANNALS OF TRANSLATIONAL MEDICINE | 10.21037/atm.2019.09.81 |
| Metagenomic next-generation sequencing aids the diagnosis of viral infections in febrile returning travellers |  | JOURNAL OF INFECTION | 10.1016/j.jinf.2019.08.003 |
| Value of mNGS in sonication fluid for the diagnosis of periprosthetic joint infection |  | ARTHROPLASTY | 10.1186/s42836-019-0006-4 |
| Metagenomic analysis using next-generation sequencing of pathogens in bronchoalveolar lavage fluid from pediatric patients with respiratory failure |  | SCIENTIFIC REPORTS | 10.1038/s41598-019-49372-x |
| The Feasibility of Metagenomic Next-Generation Sequencing to Identify Pathogens Causing Tuberculous Meningitis in Cerebrospinal Fluid |  | FRONTIERS IN MICROBIOLOGY | 10.3389/fmicb.2019.01993 |
| Metagenomic Next-Generation Sequencing of the 2014 Ebola Virus Disease Outbreak in the Democratic Republic of the Congo |  | JOURNAL OF CLINICAL MICROBIOLOGY | 10.1128/JCM.00827-19 |
| Metagenomic next-generation sequencing diagnosis of peripheral pulmonary infectious lesions through virtual navigation, radial EBUS, ultrathin bronchoscopy, and ROSE |  | JOURNAL OF INTERNATIONAL MEDICAL RESEARCH | 10.1177/0300060519866953 |
| Diagnosis of osteoarticular tuberculosis via metagenomic next-generation sequencing: A case report | CASE | EXPERIMENTAL AND THERAPEUTIC MEDICINE | 10.3892/etm.2019.7655 |
| Metagenomic Next-Generation Sequencing Reveal Presence of a Novel Ungulate Bocaparvovirus in Alpacas |  | VIRUSES-BASEL | 10.3390/v11080701 |
| Apparent performance of metagenomic next-generation sequencing in the diagnosis of cryptococcal meningitis: a descriptive study |  | JOURNAL OF MEDICAL MICROBIOLOGY | 10.1099/jmm.0.000994 |
| Metagenomic next-generation sequencing of samples from pediatric febrile illness in Tororo, Uganda |  | PLOS ONE | 10.1371/journal.pone.0218318 |
| The diagnostic value of metagenomic next-generation sequencing for identifying Streptococcus pneumoniae in paediatric bacterial meningitis |  | BMC INFECTIOUS DISEASES | 10.1186/s12879-019-4132-y |
| ANASTASIA: An Automated Metagenomic Analysis Pipeline for Novel Enzyme Discovery Exploiting Next Generation Sequencing Data |  | FRONTIERS IN GENETICS | 10.3389/fgene.2019.00469 |
| Metagenomic next-generation sequencing in clinical microbiology |  | INDIAN JOURNAL OF MEDICAL MICROBIOLOGY | 10.4103/ijmm.IJMM_19_401 |
| METAGENOMIC NEXT-GENERATION SEQUENCING REVEALS MIAMIENSIS AVIDUS (CILIOPHORA: SCUTICOCILIATIDA) IN THE 2017 EPIZOOTIC OF LEOPARD SHARKS (TRIAKIS SEMIFASCIATA) IN SAN FRANCISCO BAY, CALIFORNIA, USA |  | JOURNAL OF WILDLIFE DISEASES | 10.7589/2018-04-097 |
| Metagenomic data of fungal community in Kongsfjorden, Arctic using Illumina next generation sequencing |  | DATA IN BRIEF | 10.1016/j.dib.2018.12.026 |
| Metagenomic next-generation sequencing as a diagnostic tool for toxoplasmic encephalitis |  | ANNALS OF CLINICAL MICROBIOLOGY AND ANTIMICROBIALS | 10.1186/s12941-018-0298-1 |
| Microbiological Diagnostic Performance of Metagenomic Next-generation Sequencing When Applied to Clinical Practic |  | CLINICAL INFECTIOUS DISEASES | 10.1093/cid/ciy693 |
| Metagenomic study of bacterial microbiota in persistent endodontic infections using Next-generation sequencing |  | INTERNATIONAL ENDODONTIC JOURNAL | 10.1111/iej.12953 |
| Metagenomic Next-Generation Sequencing Reveals Individual Composition and Dynamics of Anelloviruses during Autologous Stem Cell Transplant Recipient Management |  | VIRUSES-BASEL | 10.3390/v10110633 |
| Quality control implementation for universal characterization of DNA and RNA viruses in clinical respiratory samples using single metagenomic next-generation sequencing workflow |  | BMC INFECTIOUS DISEASES | 10.1186/s12879-018-3446-5 |
| Development and Optimization of Metagenomic Next-Generation Sequencing Methods for Cerebrospinal Fluid Diagnostics |  | JOURNAL OF CLINICAL MICROBIOLOGY | 10.1128/JCM.00472-18 |
| Chronic Meningitis Investigated via Metagenomic Next-Generation Sequencing |  | JAMA NEUROLOGY | 10.1001/jamaneurol.2018.0463 |
| Detection of Pulmonary Infectious Pathogens From Lung Biopsy Tissues by Metagenomic Next-Generation Sequencing |  | FRONTIERS IN CELLULAR AND INFECTION MICROBIOLOGY | 10.3389/fcimb.2018.00205 |
| Cost-benefit analysis of introducing next-generation sequencing (metagenomic) pathogen testing in the setting of pyrexia of unknown origin |  | PLOS ONE | 10.1371/journal.pone.0194648 |
| Understanding the Promises and Hurdles of Metagenomic Next-Generation Sequencing as a Diagnostic Tool for Infectious Diseases |  | CLINICAL INFECTIOUS DISEASES | 10.1093/cid/cix881 |
| Neurobrucellosis: Unexpected Answer From Metagenomic Next-Generation Sequencing |  | JOURNAL OF THE PEDIATRIC INFECTIOUS DISEASES SOCIETY | 10.1093/jpids/piw066 |
| Viral Surveillance in Serum Samples From Patients With Acute Liver Failure By Metagenomic Next-Generation Sequencing |  | CLINICAL INFECTIOUS DISEASES | 10.1093/cid/cix596 |
| A viral metagenomic approach on a non-metagenomic experiment: Mining next generation sequencing datasets from pig DNA identified several porcine parvoviruses for a retrospective evaluation of viral infections |  | PLOS ONE | 10.1371/journal.pone.0179462 |
| Rule-Out Outbreak: 24-Hour Metagenomic Next-Generation Sequencing for Characterizing Respiratory Virus Source for Infection Prevention |  | JOURNAL OF THE PEDIATRIC INFECTIOUS DISEASES SOCIETY | 10.1093/jpids/pix019 |
| Validation of Metagenomic Next-Generation Sequencing Tests for Universal Pathogen Detection |  | ARCHIVES OF PATHOLOGY & LABORATORY MEDICINE | 10.5858/arpa.2016-0539-RA |
| Rapid Metagenomic Next-Generation Sequencing during an Investigation of Hospital-Acquired Human Parainfluenza Virus 3 Infections |  | JOURNAL OF CLINICAL MICROBIOLOGY | 10.1128/JCM.01881-16 |
| Coinfections of Zika and Chikungunya Viruses in Bahia, Brazil, Identified by Metagenomic Next-Generation Sequencing |  | JOURNAL OF CLINICAL MICROBIOLOGY | 10.1128/JCM.00877-16 |
| Fatal Psychrobacter sp infection in a pediatric patient with meningitis identified by metagenomic next-generation sequencing in cerebrospinal fluid |  | ARCHIVES OF MICROBIOLOGY | 10.1007/s00203-015-1168-2 |
| Culture-Independent Metagenomic Surveillance of Commercially Available Probiotics with High-Throughput Next-Generation Sequencing |  | MSPHERE | 10.1128/mSphere.00057-16 |
| MetaObtainer: A Tool for Obtaining Specified Species from Metagenomic Reads of Next-generation Sequencing |  | INTERDISCIPLINARY SCIENCES-COMPUTATIONAL LIFE SCIENCES | 10.1007/s12539-015-0281-x |
| Utility of Metagenomic Next-Generation Sequencing for Characterization of HIV and Human Pegivirus Diversity |  | PLOS ONE | 10.1371/journal.pone.0141723 |
| An ensemble strategy that significantly improves de novo assembly of microbial genomes from metagenomic next-generation sequencing data |  | NUCLEIC ACIDS RESEARCH | 10.1093/nar/gkv002 |
| Comparison of three next-generation sequencing platforms for metagenomic sequencing and identification of pathogens in blood |  | BMC GENOMICS | 10.1186/1471-2164-15-96 |
| Coupled high-throughput functional screening and next generation sequencing for identification of plant polymer decomposing enzymes in metagenomic libraries |  | FRONTIERS IN MICROBIOLOGY | 10.3389/fmicb.2013.00282 |
| Assessment of Metagenomic Assembly Using Simulated Next Generation Sequencing Data |  | PLOS ONE | 10.1371/journal.pone.0031386 |
| Next generation sequencing and bioinformatic bottlenecks: the current state of metagenomic data analysis |  | CURRENT OPINION IN BIOTECHNOLOGY | 10.1016/j.copbio.2011.11.013 |
| Next-generation sequencing and metagenomic analysis: a universal diagnostic tool in plant virology |  | MOLECULAR PLANT PATHOLOGY | 10.1111/J.1364-3703.2009.00545.X |
